# Supplementary figures and images for: Effects of Drosophila melanogaster regular exercise and apolipoprotein B knockdown on abnormal heart rhythm induced by a high-fat diet
Source: PLoS One. 2022 Jun 3;17(6):e0262471. doi: 10.1371/journal.pone.0262471 (PMC9165823; doi:10.1371/journal.pone.0262471)

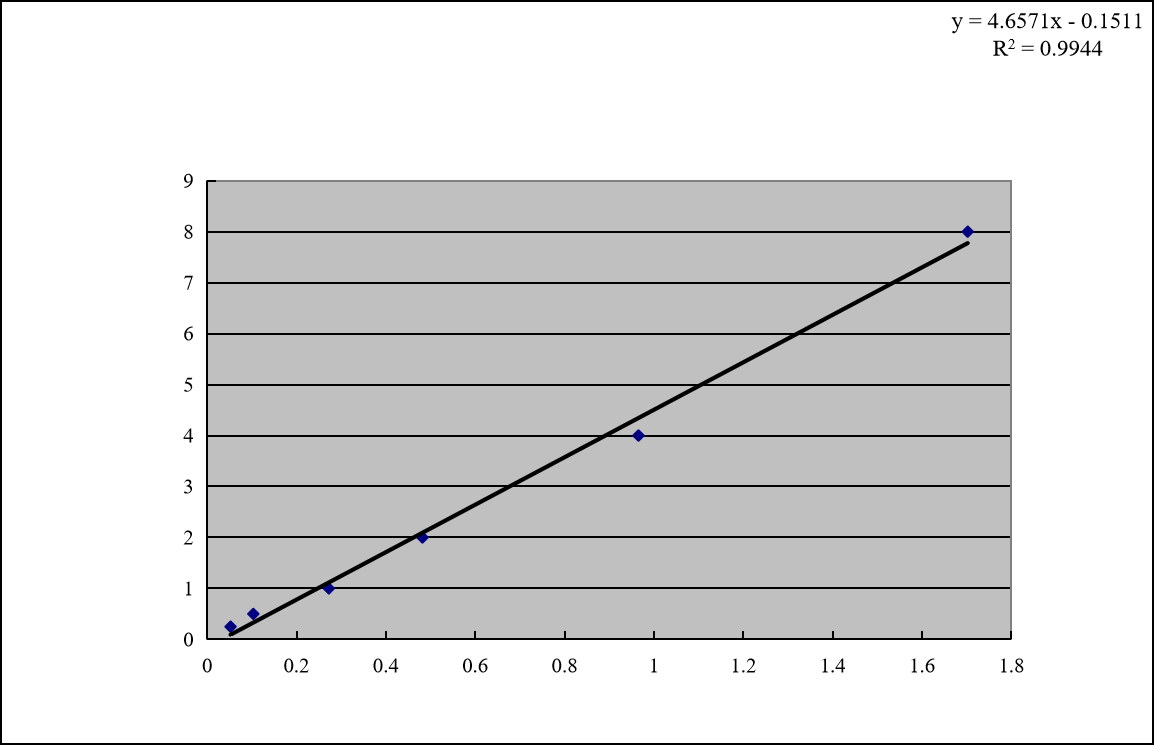

Supplement: S1 Fig — (TIF) [file pone.0262471.s001.tif]

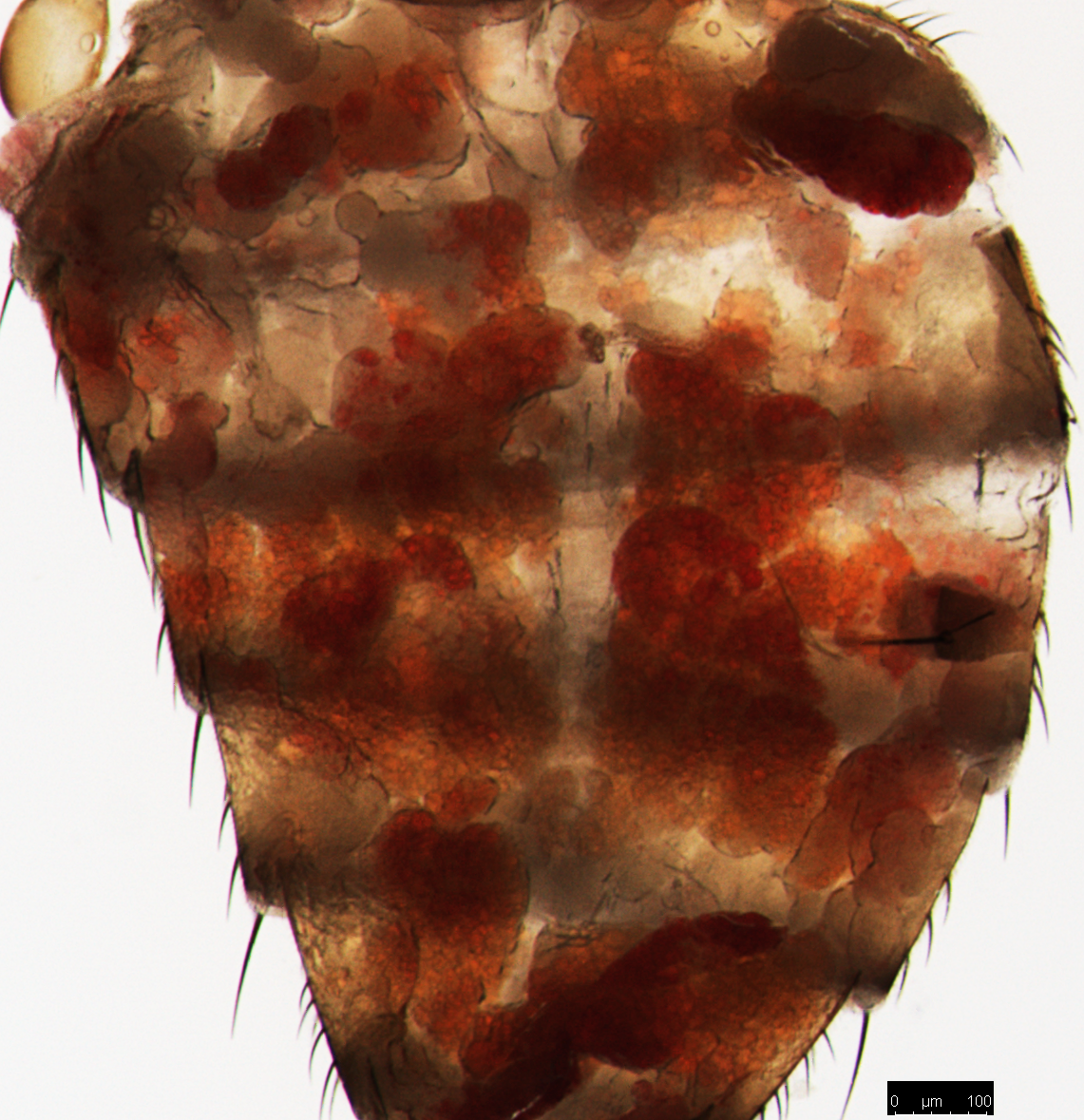

Supplement: S1 File — (ZIP) [file pone.0262471.s004.zip › S1-File-ORO/ORO/Fig1/HFD/1.tif]

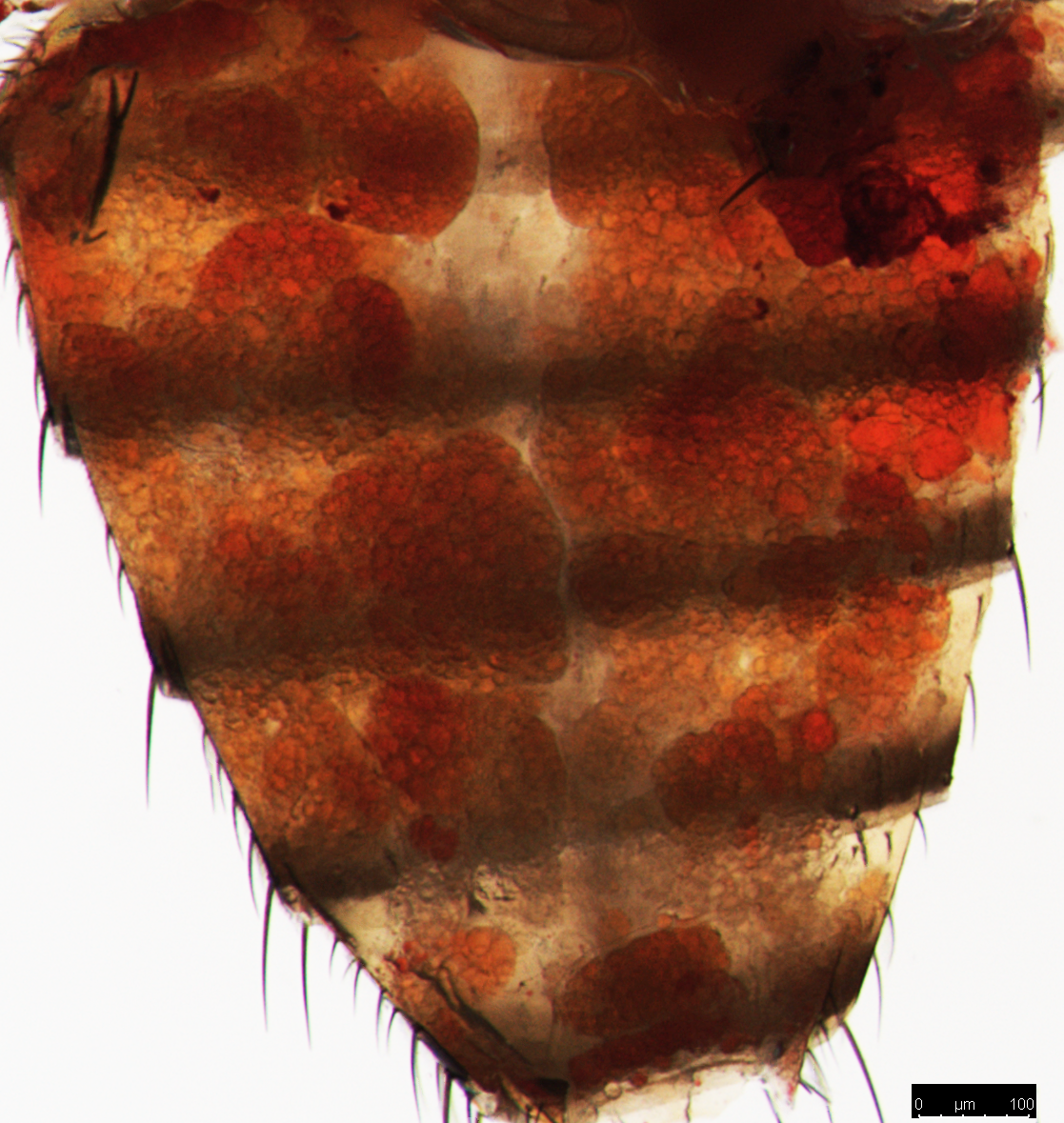

Supplement: S1 File — (ZIP) [file pone.0262471.s004.zip › S1-File-ORO/ORO/Fig1/HFD/2.tif]

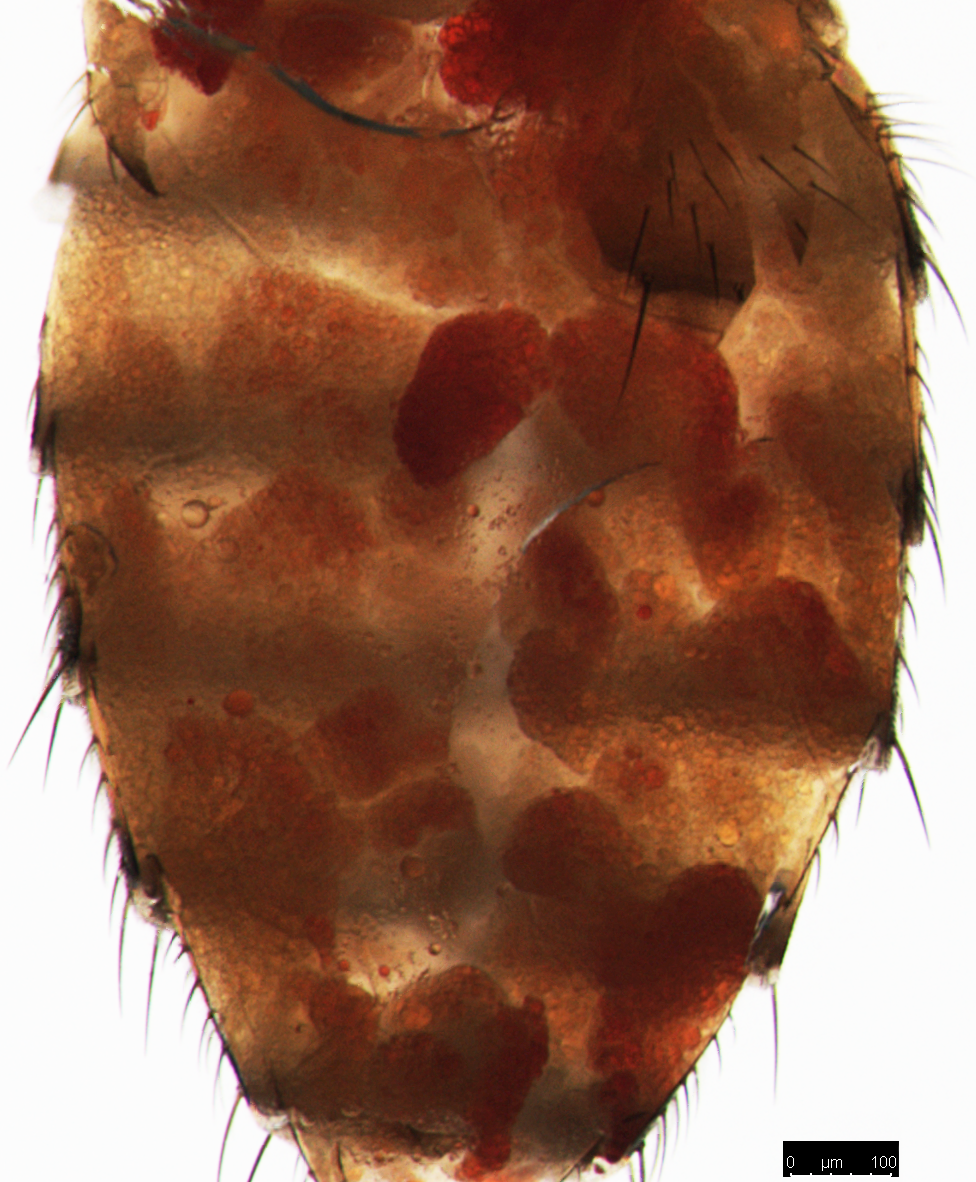

Supplement: S1 File — (ZIP) [file pone.0262471.s004.zip › S1-File-ORO/ORO/Fig1/HFD/3.tif]

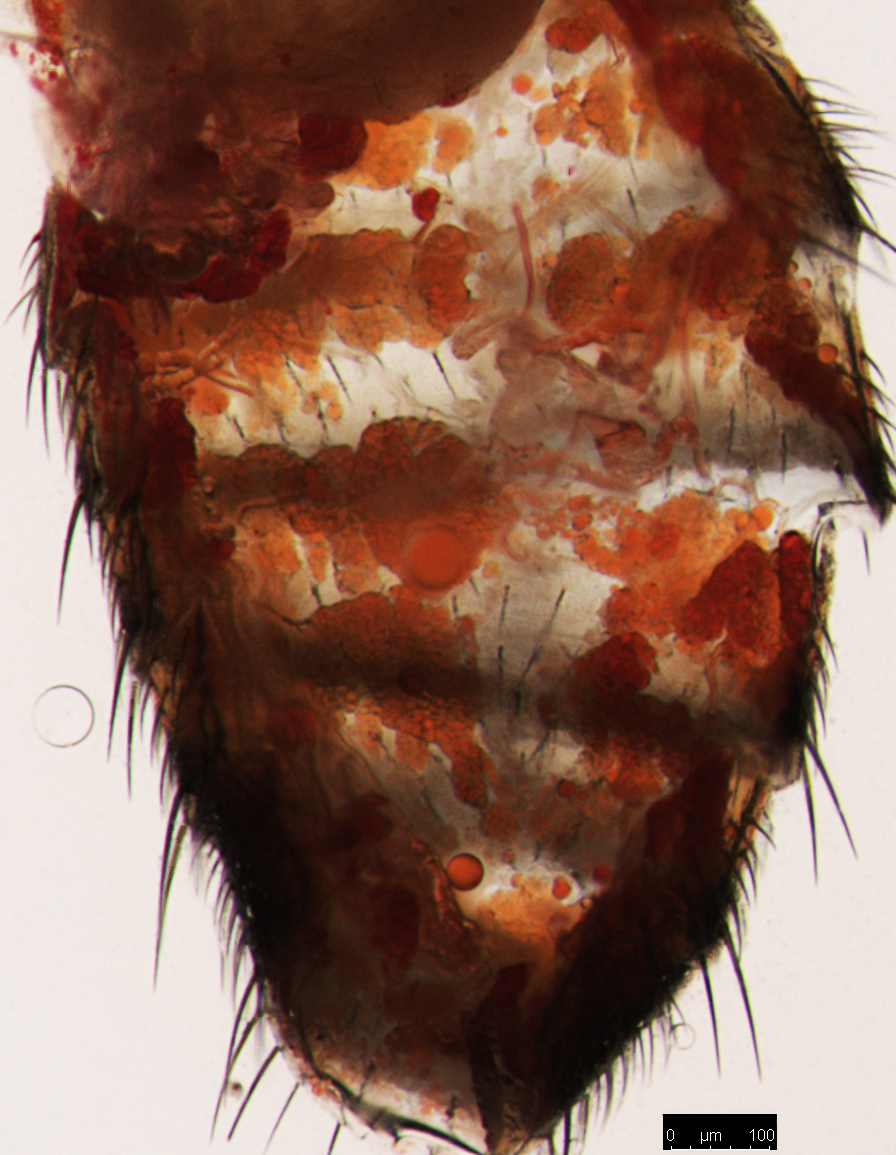

Supplement: S1 File — (ZIP) [file pone.0262471.s004.zip › S1-File-ORO/ORO/Fig1/HFD/4.tif]

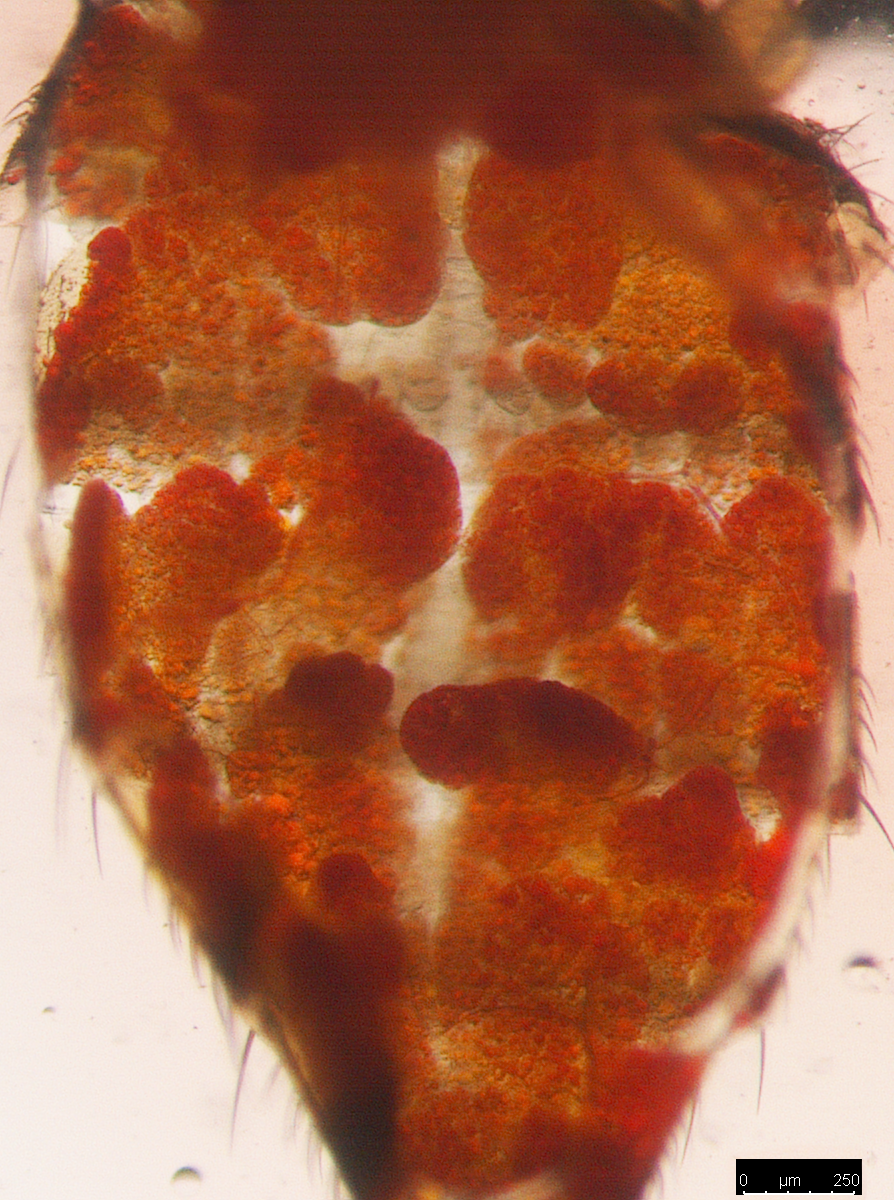

Supplement: S1 File — (ZIP) [file pone.0262471.s004.zip › S1-File-ORO/ORO/Fig1/HFD/HFD2.tif]

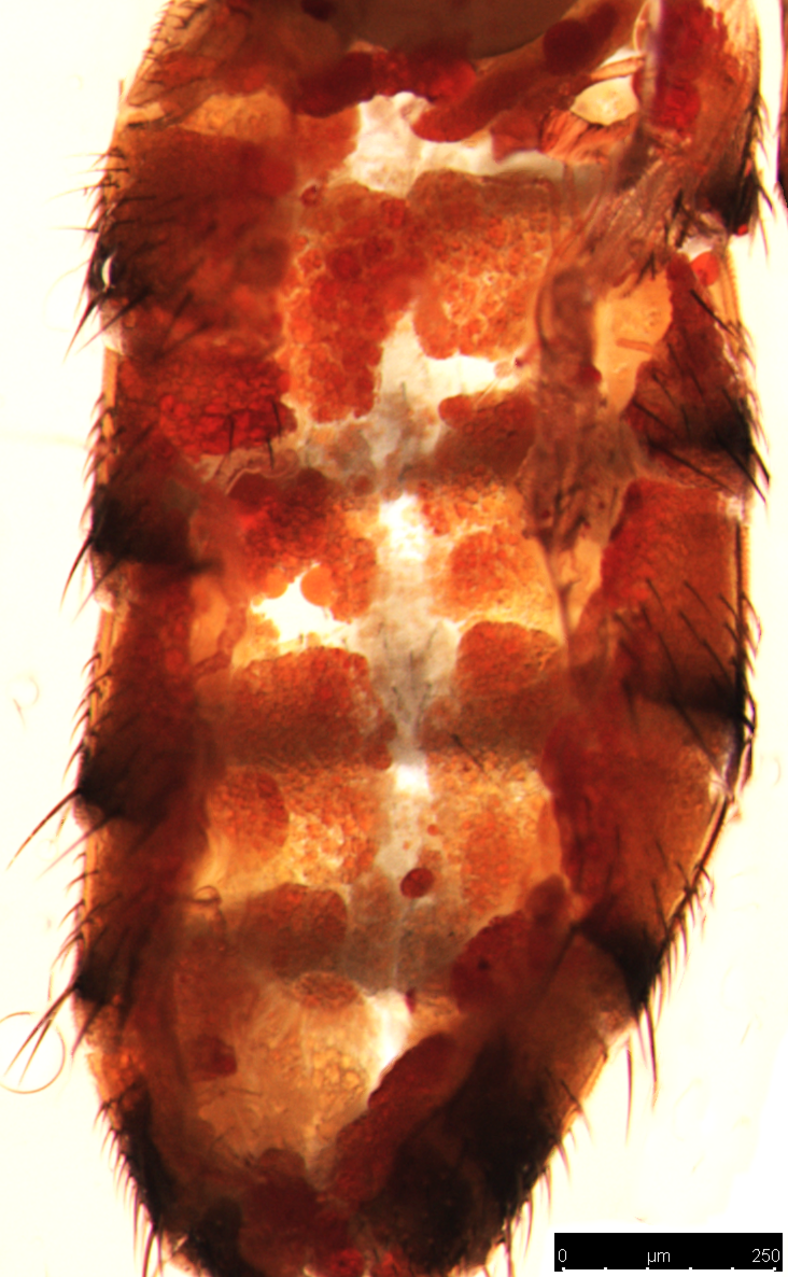

Supplement: S1 File — (ZIP) [file pone.0262471.s004.zip › S1-File-ORO/ORO/Fig1/NF/1.tif]

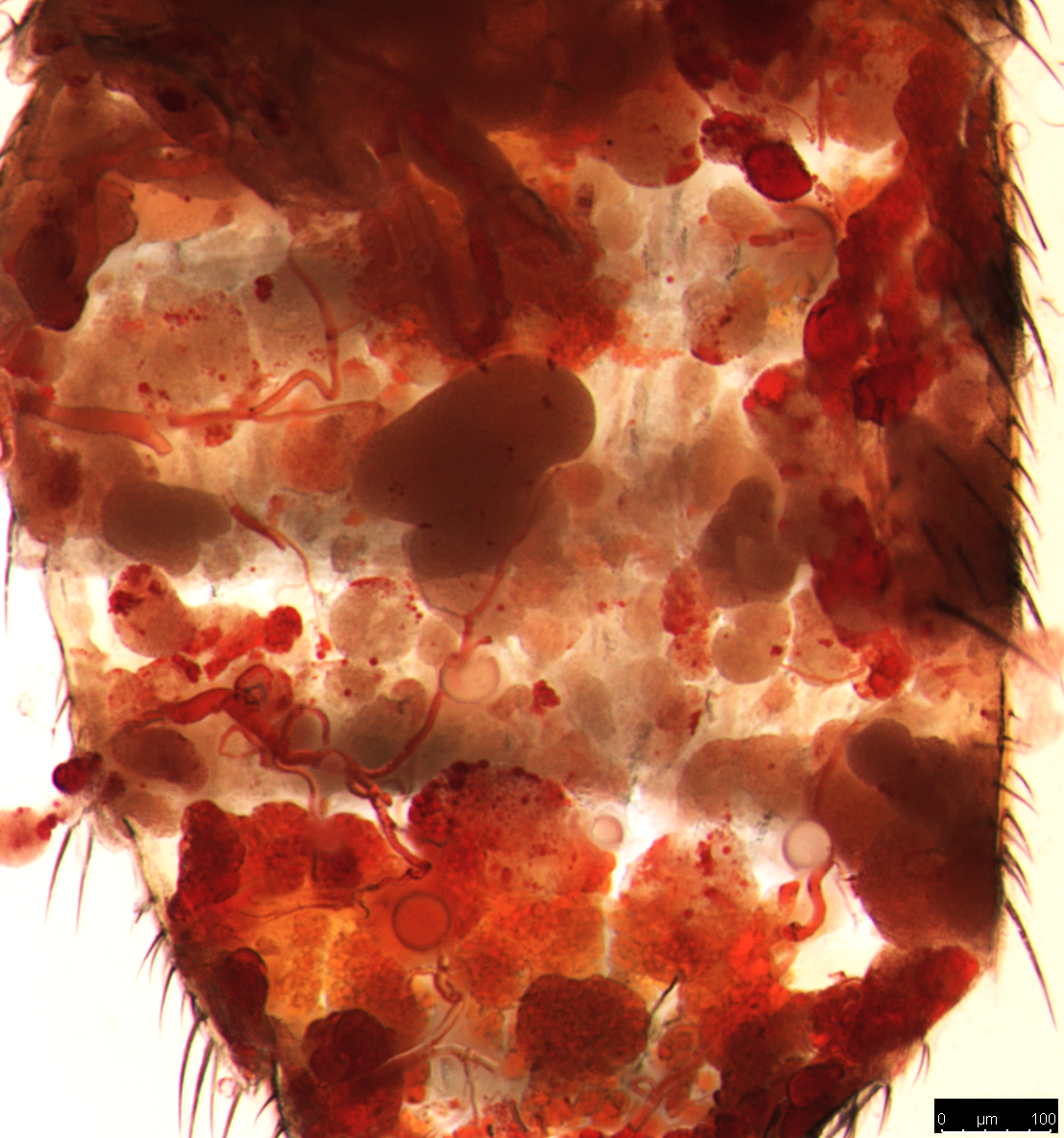

Supplement: S1 File — (ZIP) [file pone.0262471.s004.zip › S1-File-ORO/ORO/Fig1/NF/2.tif]

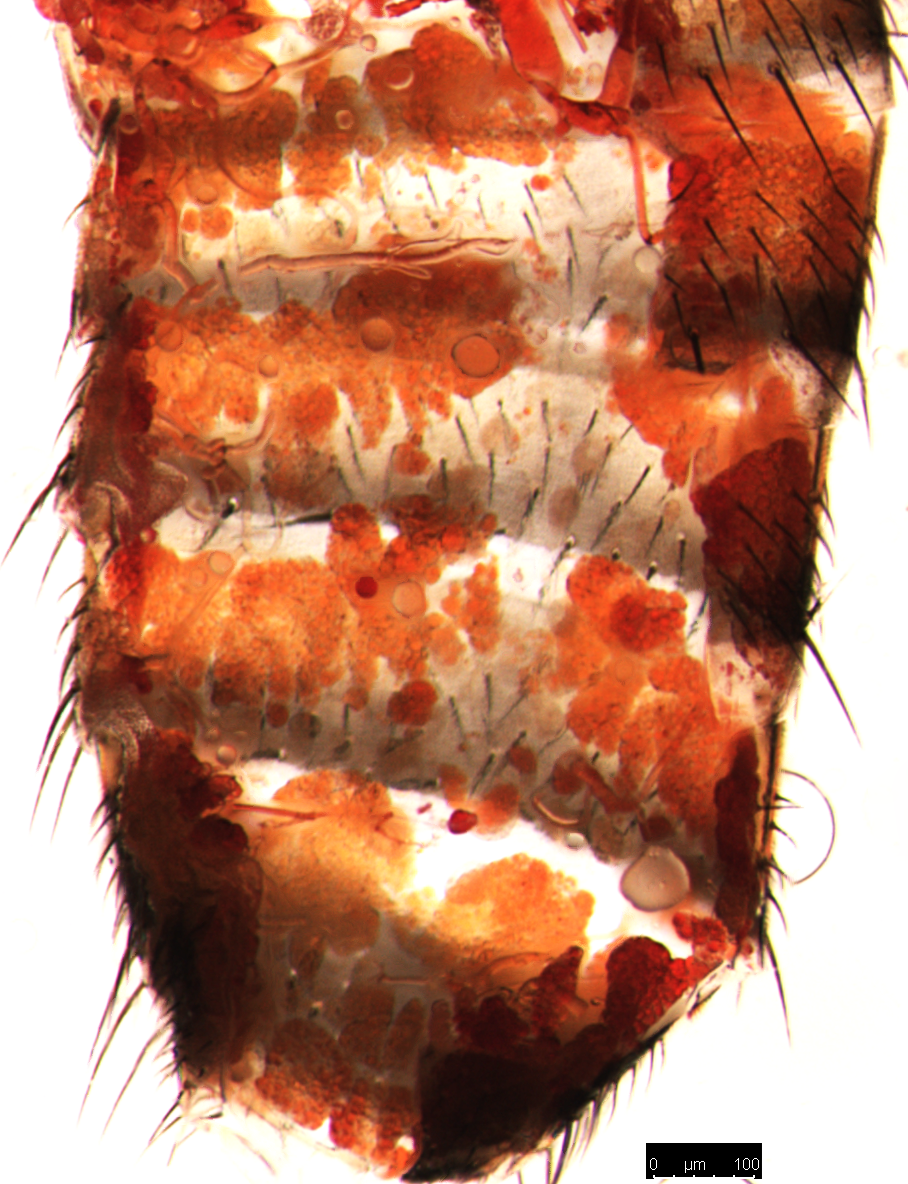

Supplement: S1 File — (ZIP) [file pone.0262471.s004.zip › S1-File-ORO/ORO/Fig1/NF/3.tif]

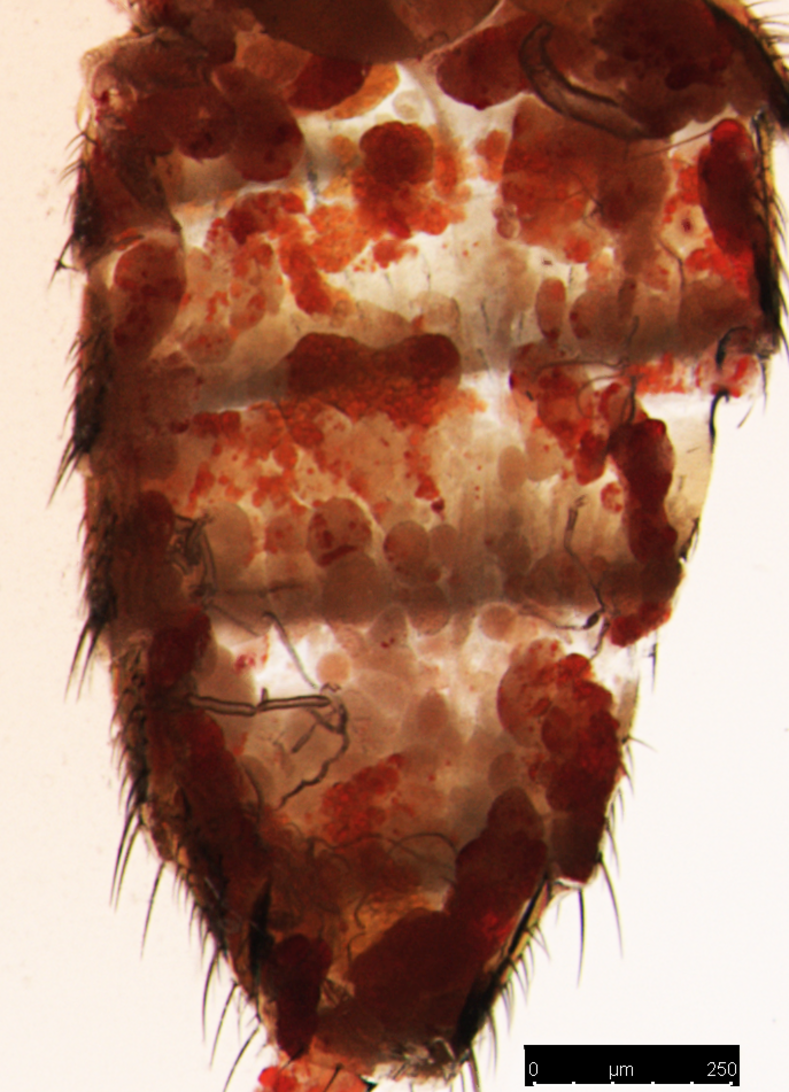

Supplement: S1 File — (ZIP) [file pone.0262471.s004.zip › S1-File-ORO/ORO/Fig1/NF/4.tif]

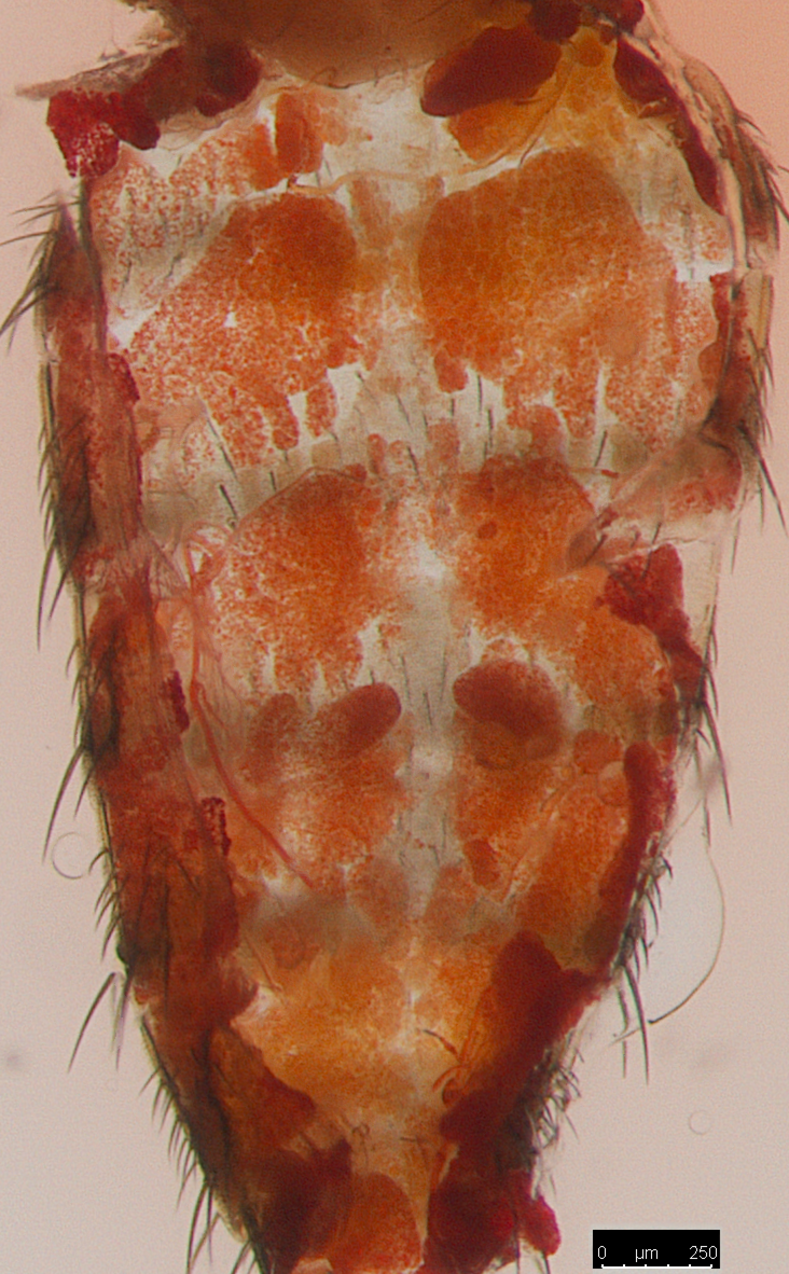

Supplement: S1 File — (ZIP) [file pone.0262471.s004.zip › S1-File-ORO/ORO/Fig1/NF/NF.tif]

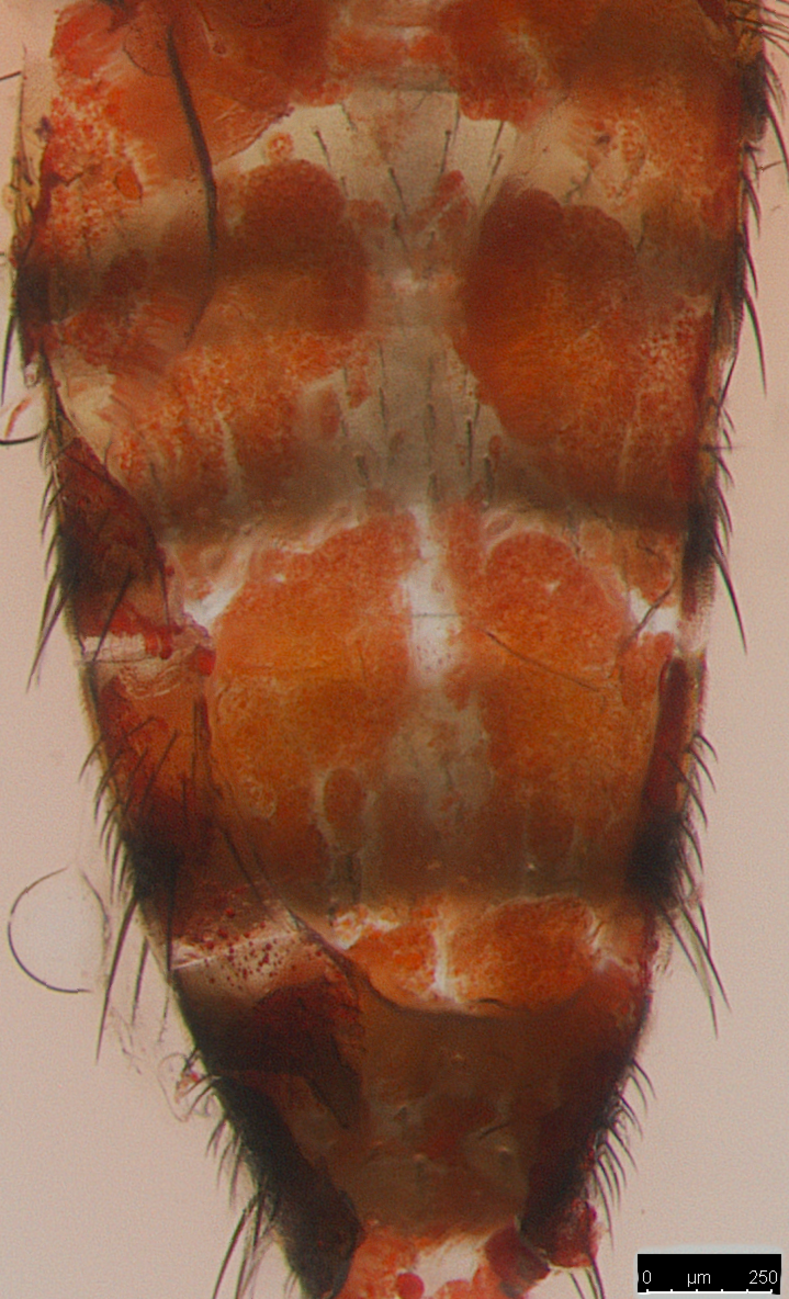

Supplement: S1 File — (ZIP) [file pone.0262471.s004.zip › S1-File-ORO/ORO/Fig2/HFD+KD/1.tif]

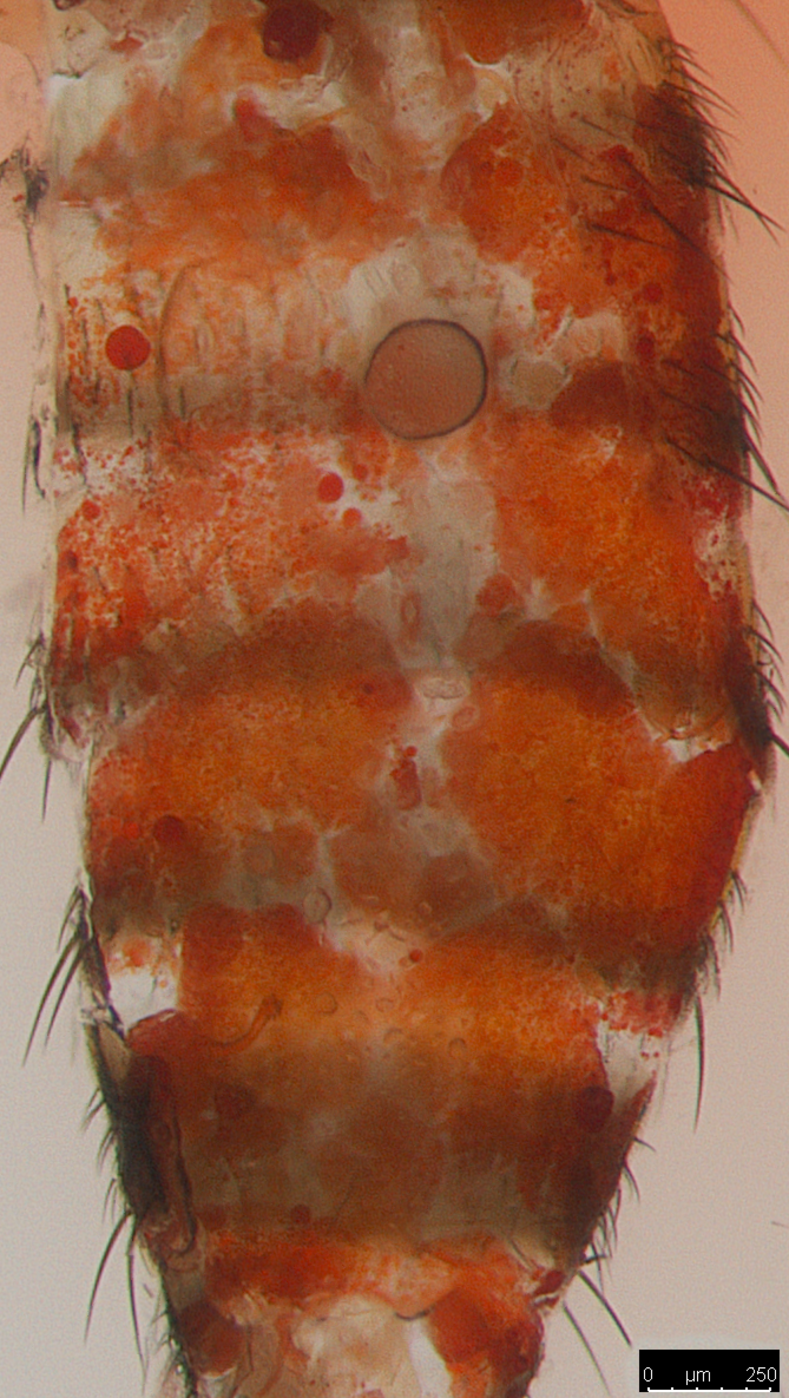

Supplement: S1 File — (ZIP) [file pone.0262471.s004.zip › S1-File-ORO/ORO/Fig2/HFD+KD/2.tif]

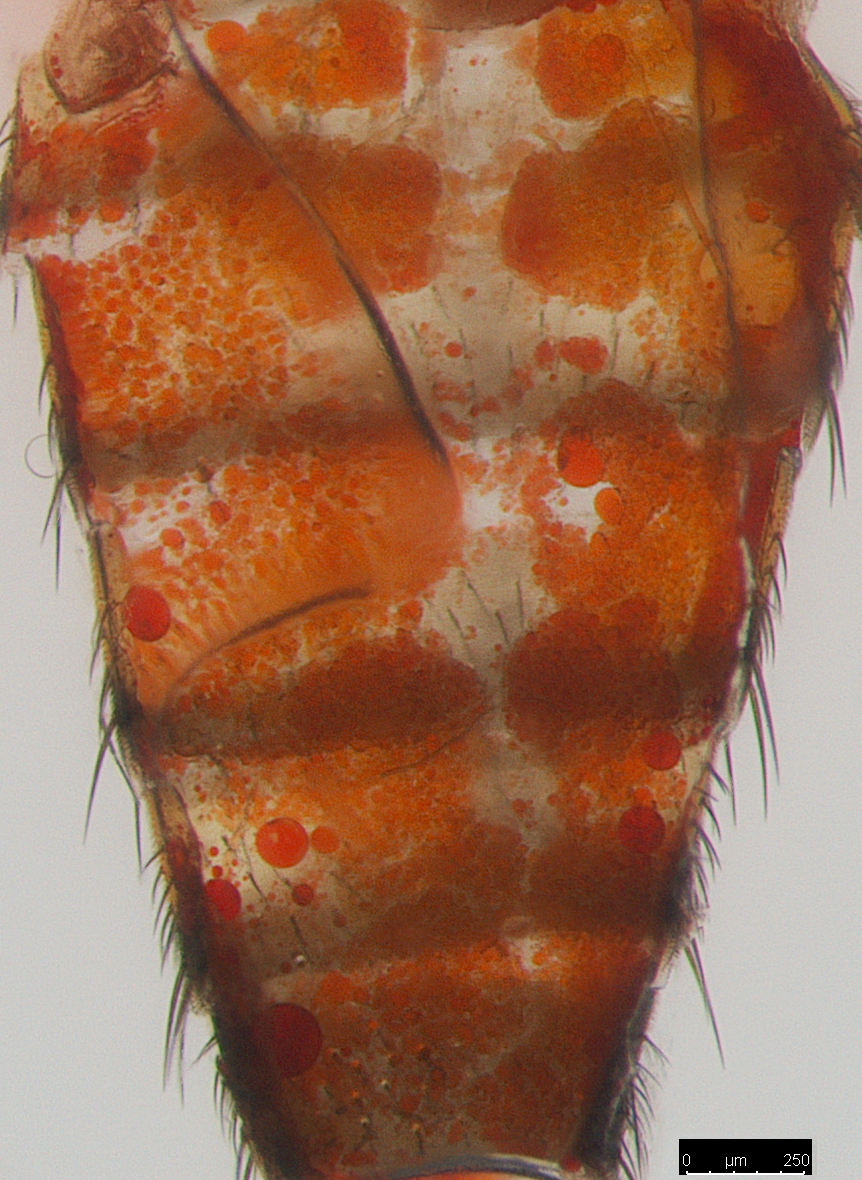

Supplement: S1 File — (ZIP) [file pone.0262471.s004.zip › S1-File-ORO/ORO/Fig2/HFD+KD/3.tif]

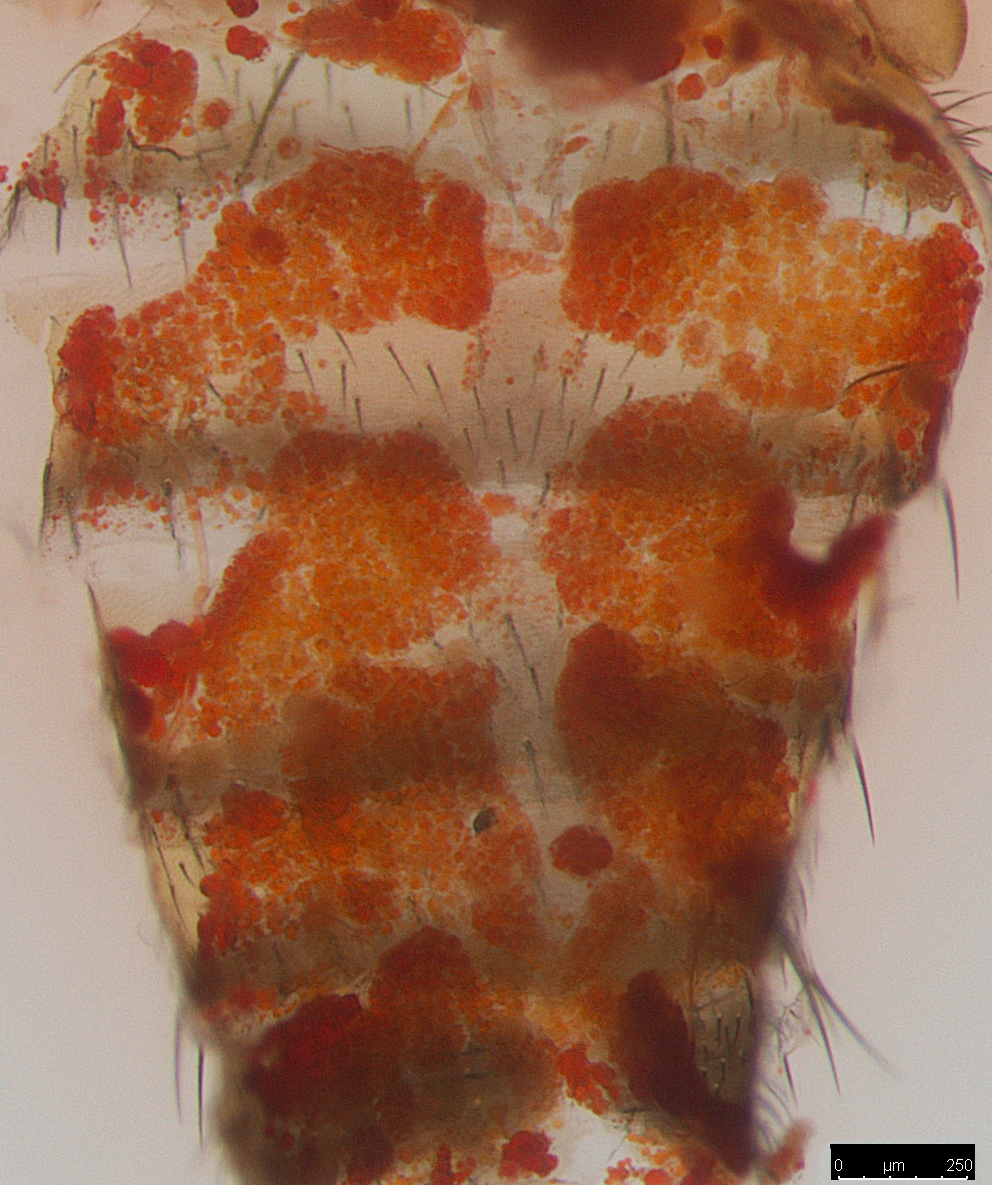

Supplement: S1 File — (ZIP) [file pone.0262471.s004.zip › S1-File-ORO/ORO/Fig2/HFD+KD/4.tif]

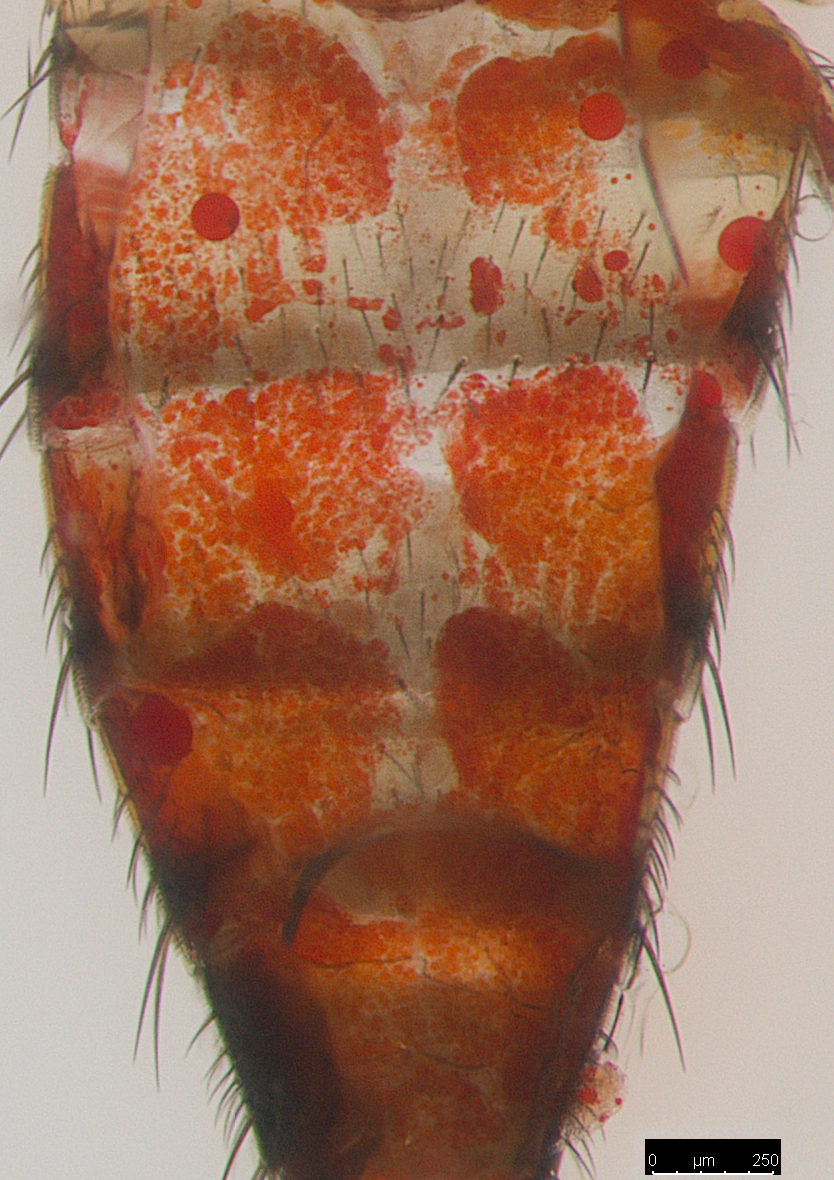

Supplement: S1 File — (ZIP) [file pone.0262471.s004.zip › S1-File-ORO/ORO/Fig2/HFD+KD/HFD+KD1.tif]

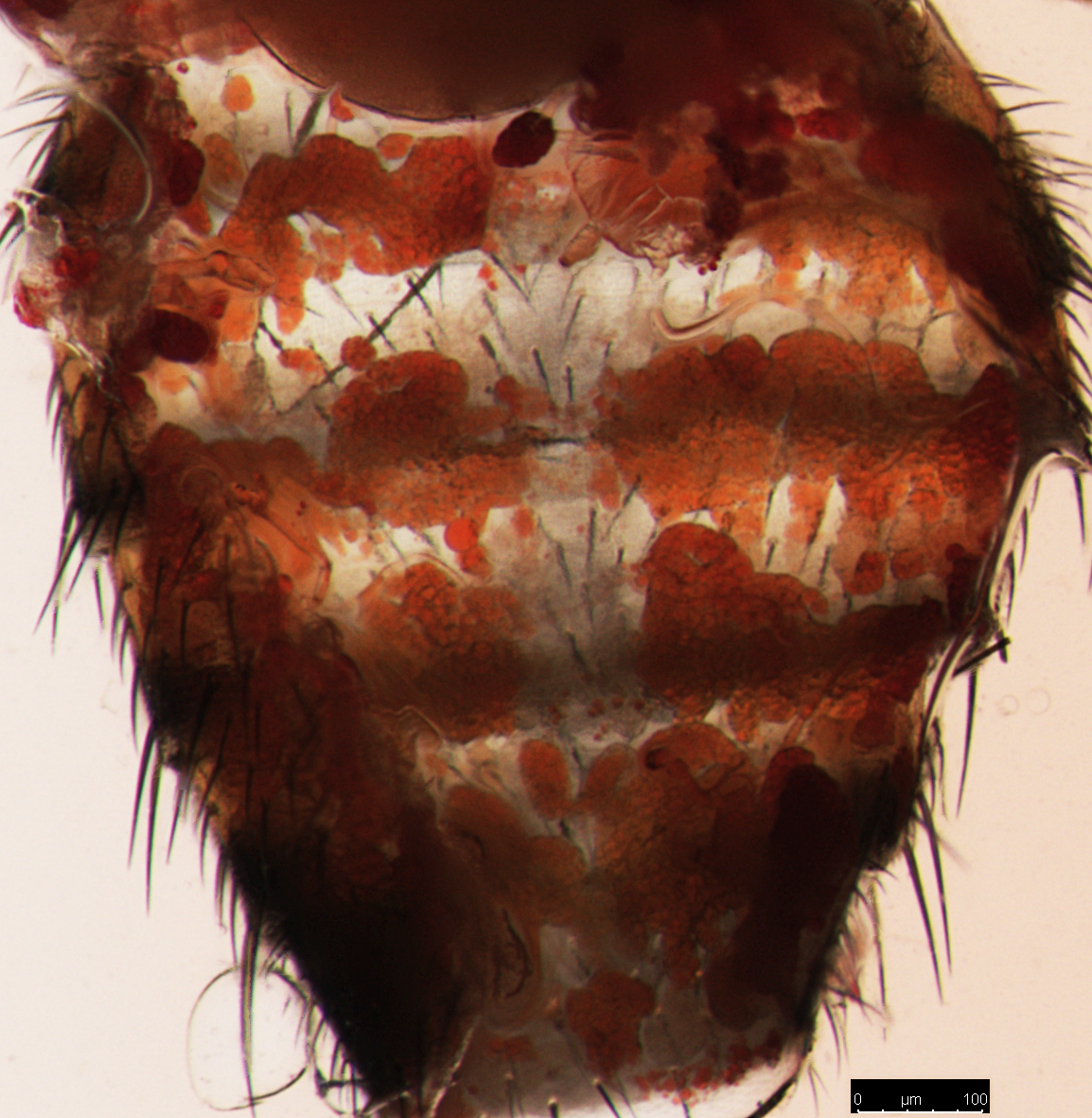

Supplement: S1 File — (ZIP) [file pone.0262471.s004.zip › S1-File-ORO/ORO/Fig2/HFD/1.tif]

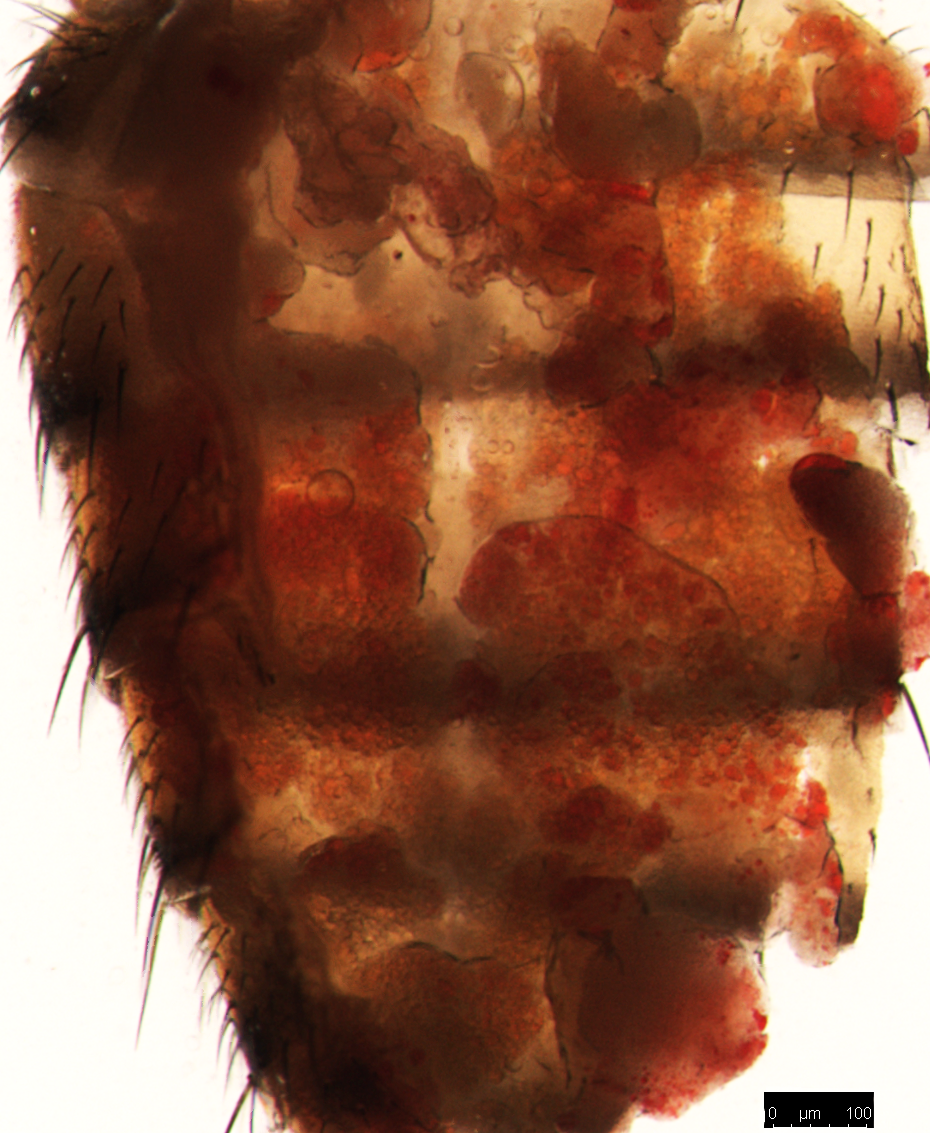

Supplement: S1 File — (ZIP) [file pone.0262471.s004.zip › S1-File-ORO/ORO/Fig2/HFD/2.tif]

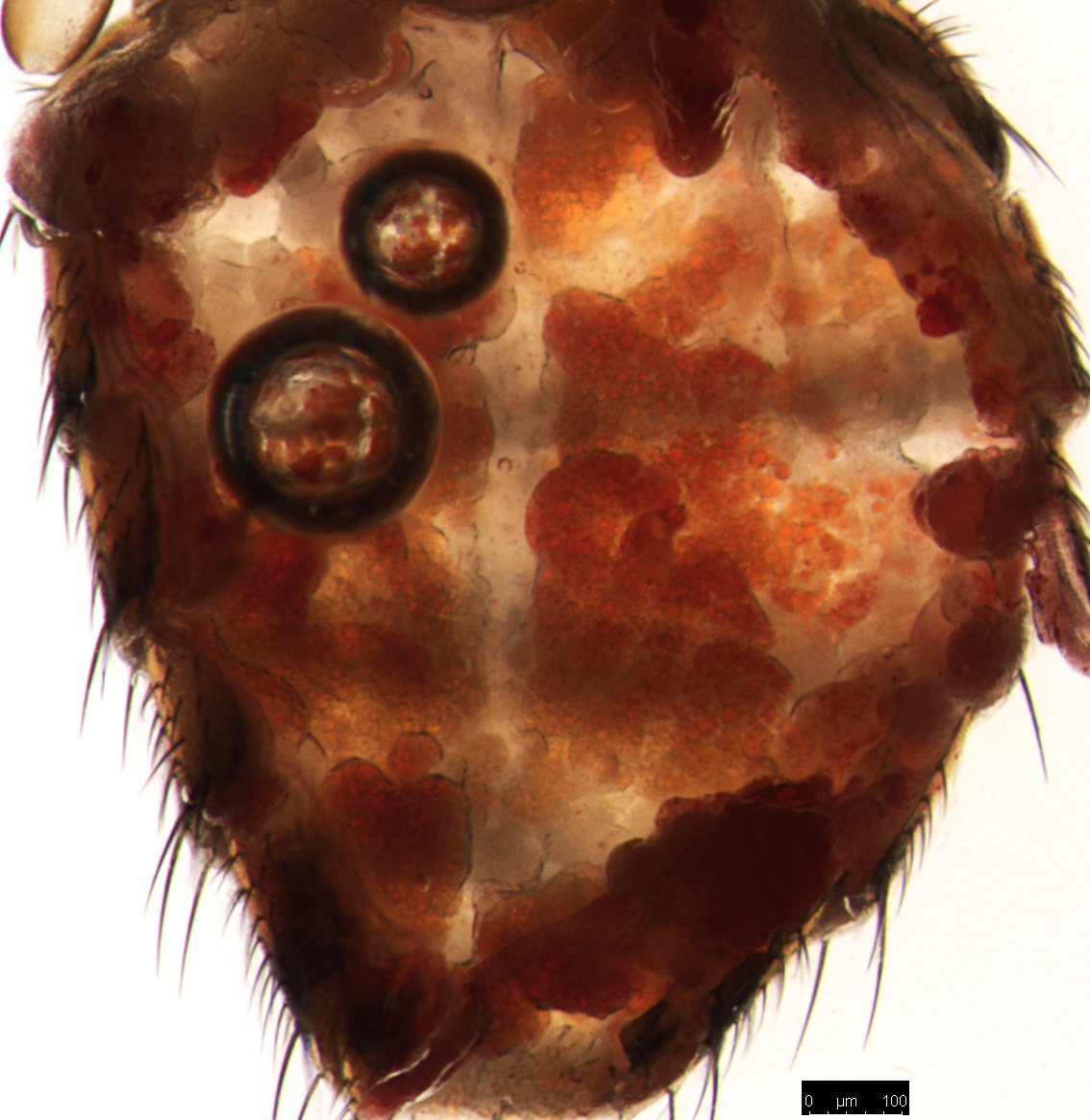

Supplement: S1 File — (ZIP) [file pone.0262471.s004.zip › S1-File-ORO/ORO/Fig2/HFD/3.tif]

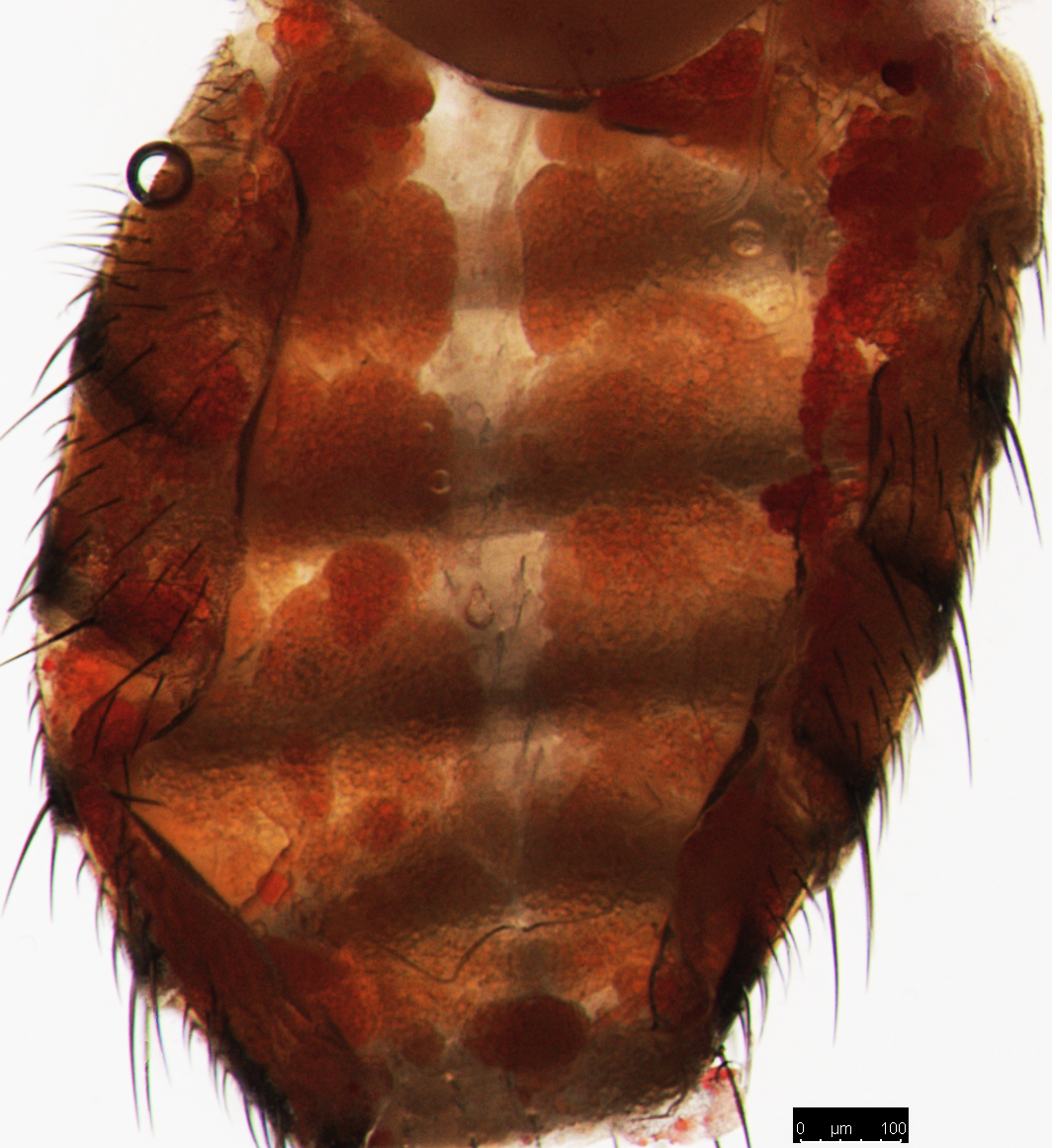

Supplement: S1 File — (ZIP) [file pone.0262471.s004.zip › S1-File-ORO/ORO/Fig2/HFD/4.tif]

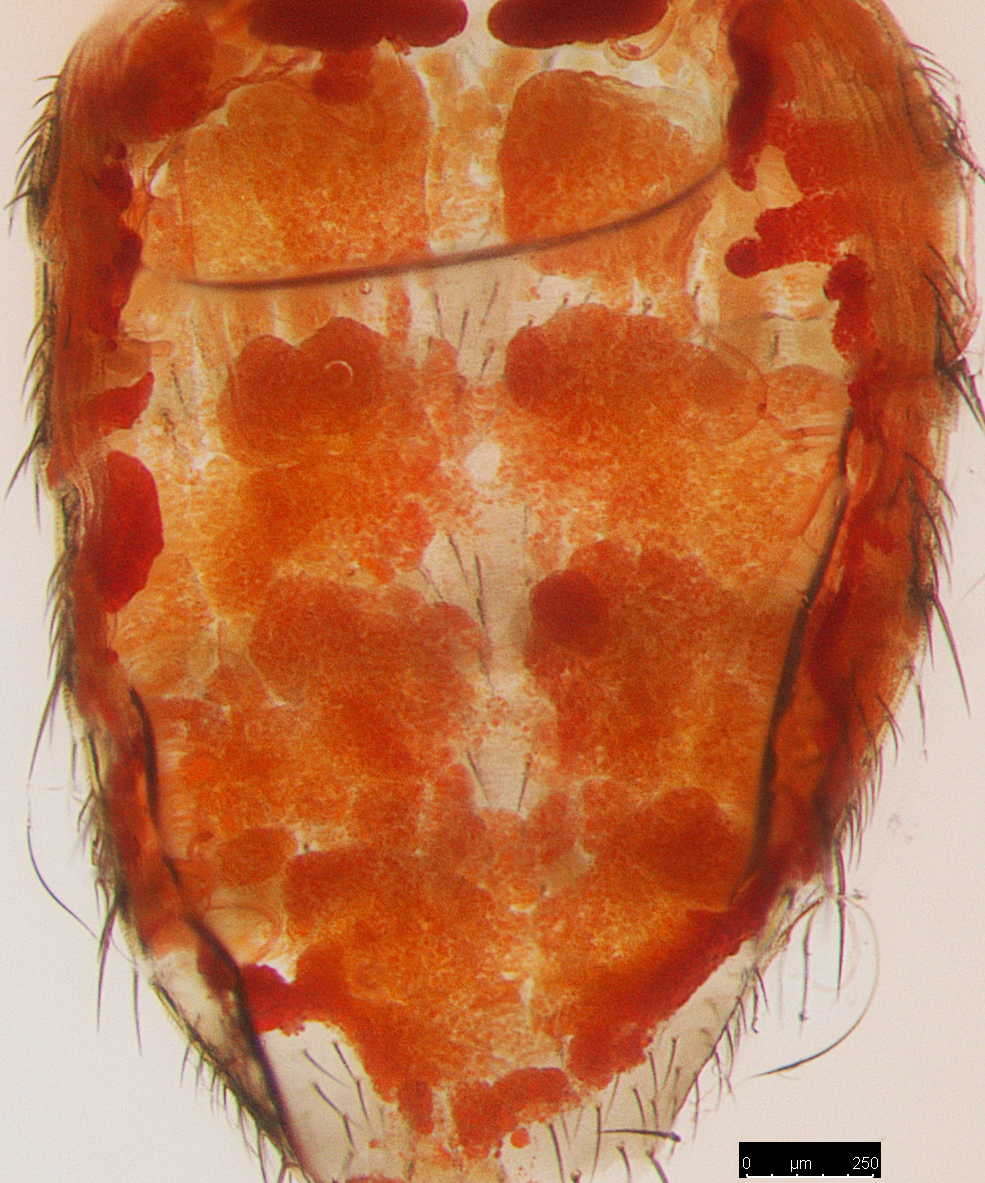

Supplement: S1 File — (ZIP) [file pone.0262471.s004.zip › S1-File-ORO/ORO/Fig2/HFD/HFD1.tif]

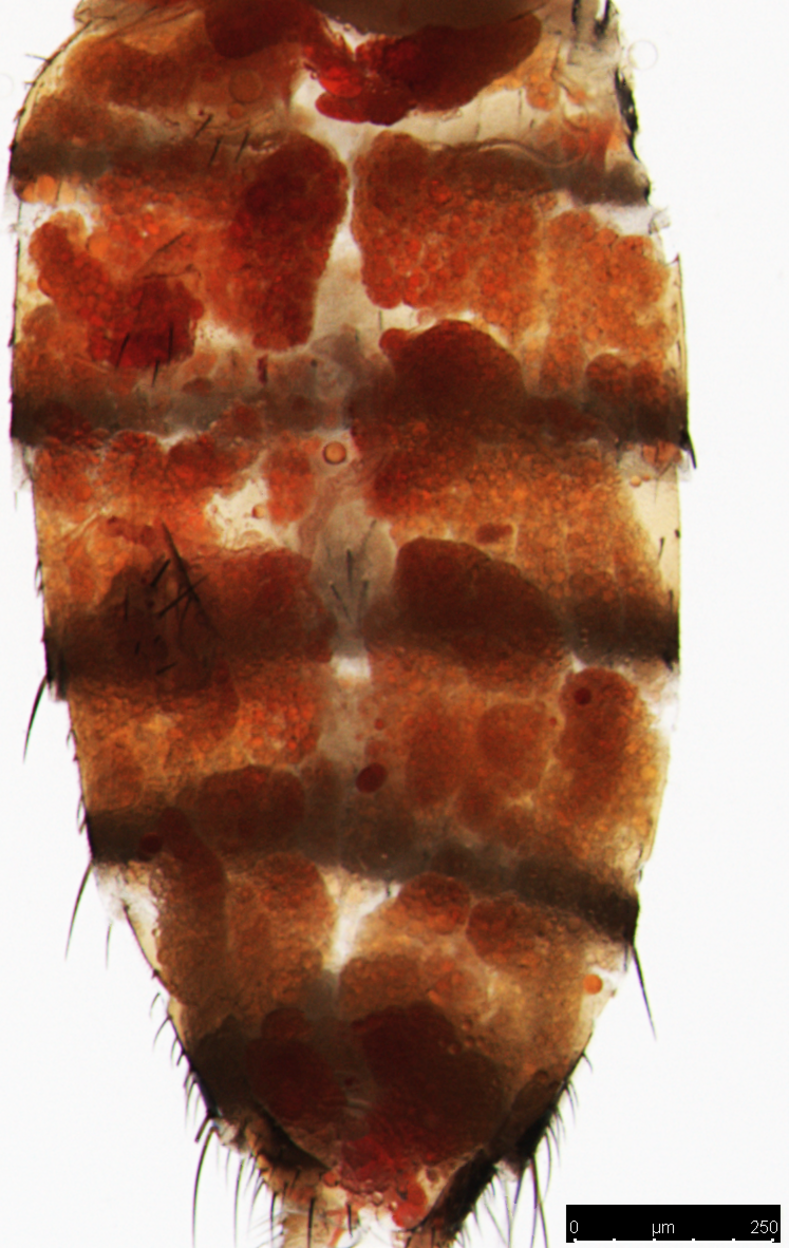

Supplement: S1 File — (ZIP) [file pone.0262471.s004.zip › S1-File-ORO/ORO/Fig3/HFD+E/1.tif]

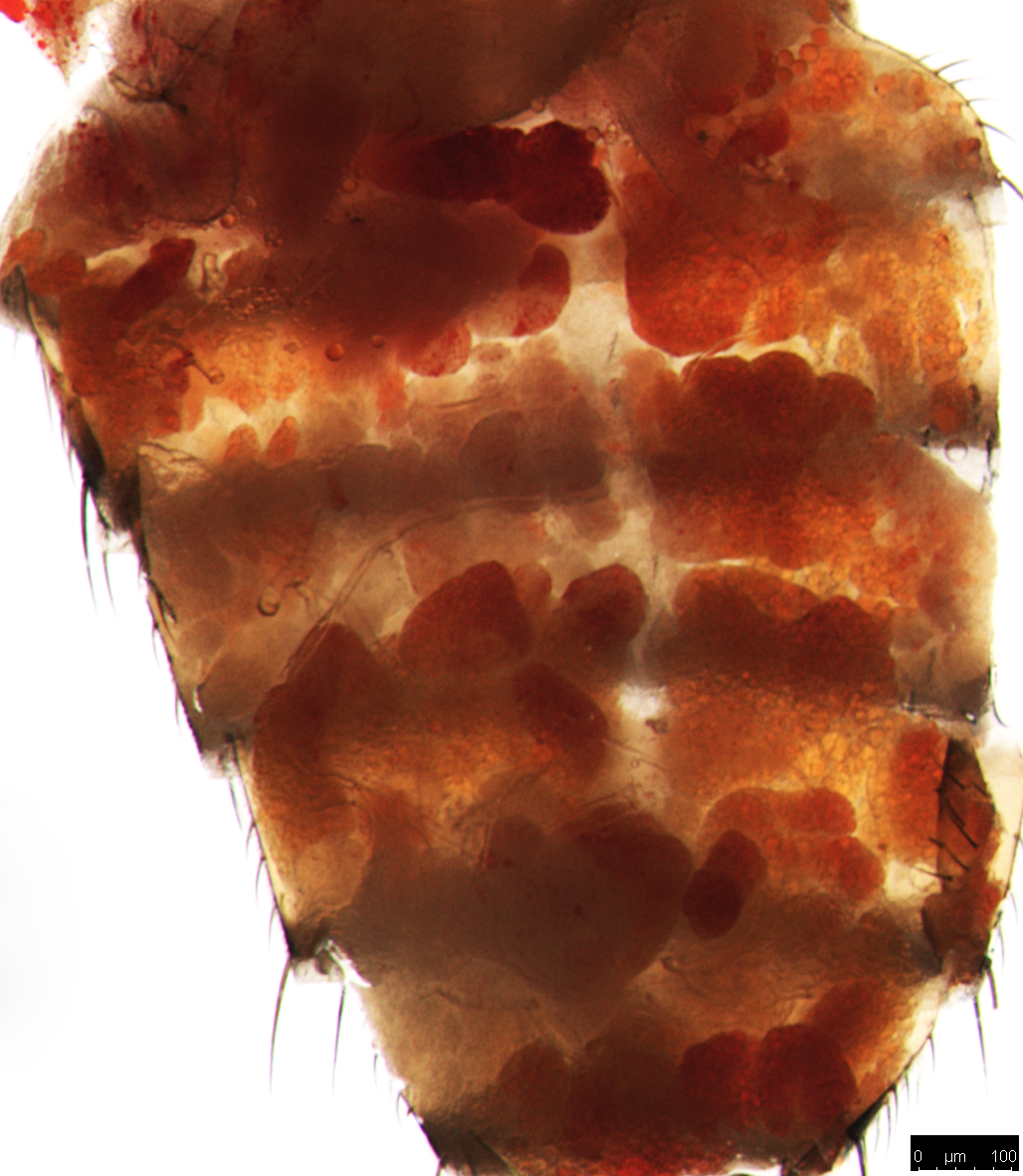

Supplement: S1 File — (ZIP) [file pone.0262471.s004.zip › S1-File-ORO/ORO/Fig3/HFD+E/2.tif]

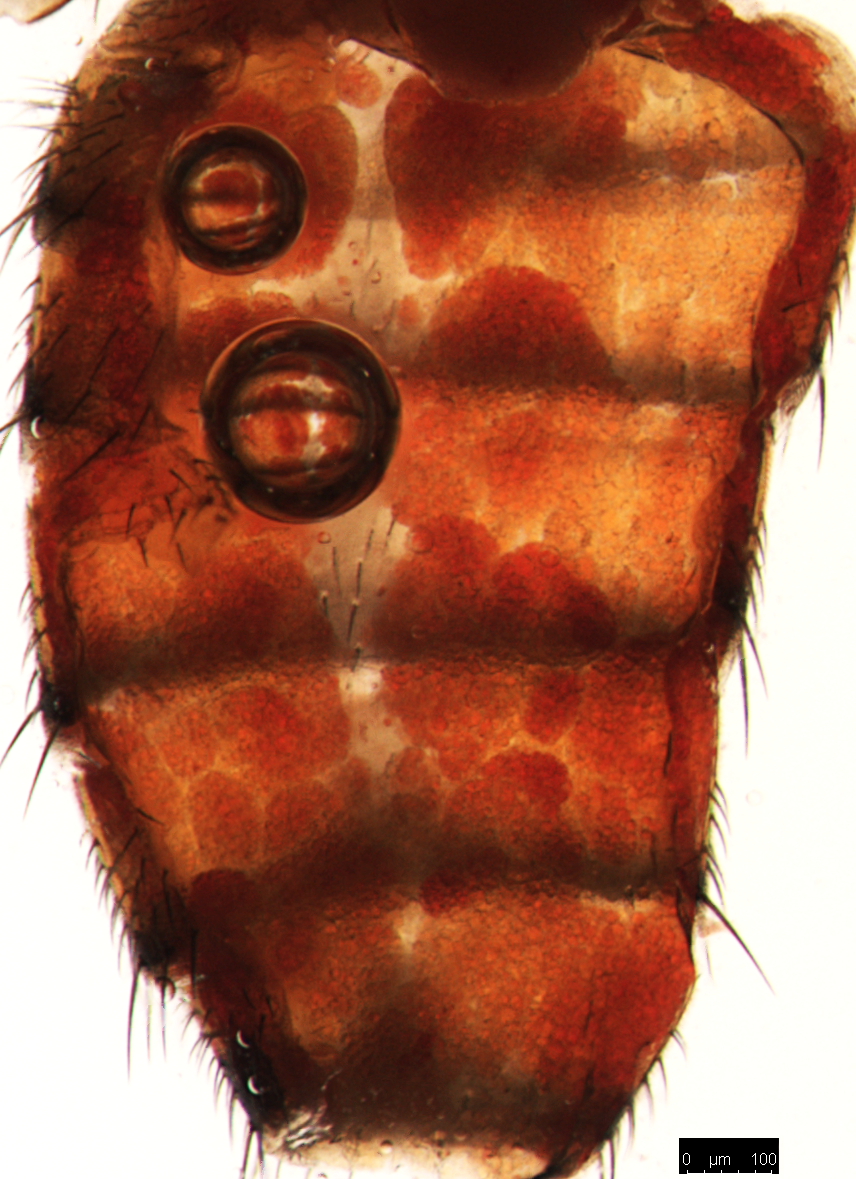

Supplement: S1 File — (ZIP) [file pone.0262471.s004.zip › S1-File-ORO/ORO/Fig3/HFD+E/3.tif]

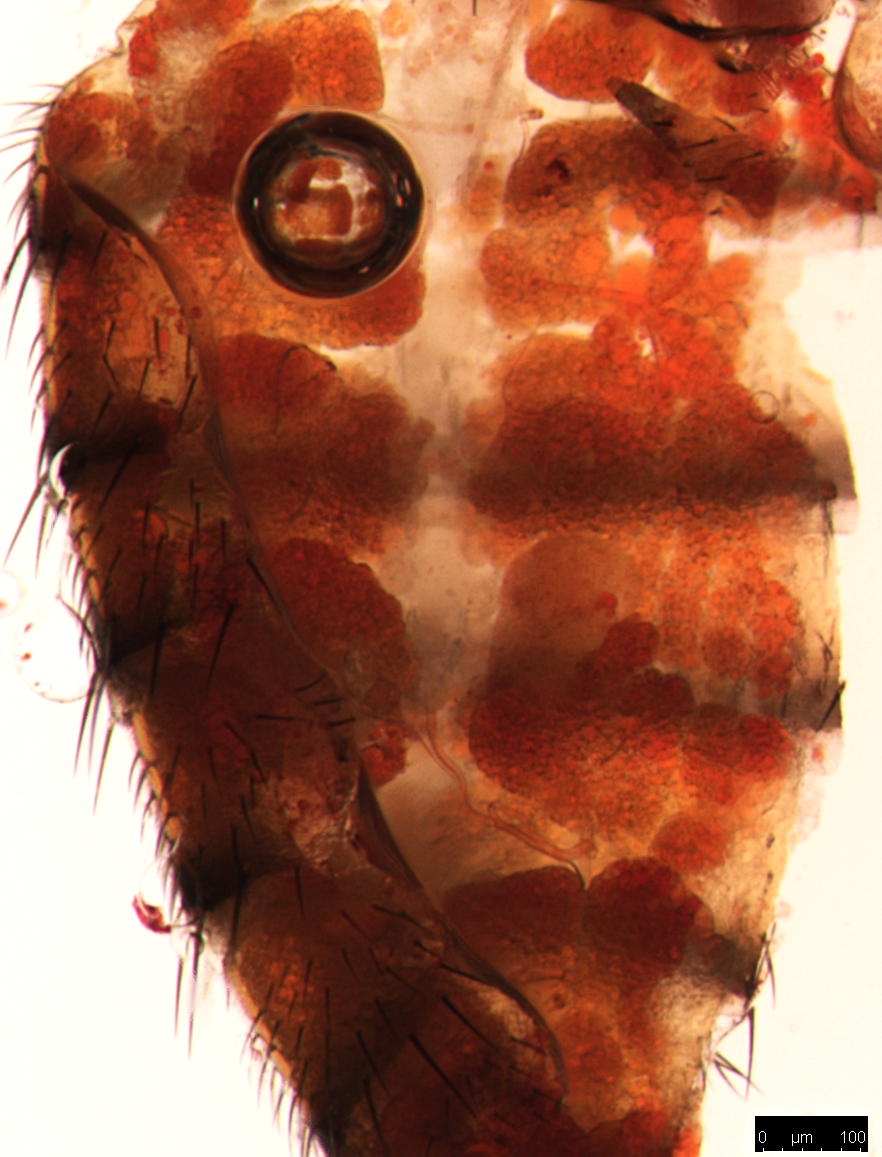

Supplement: S1 File — (ZIP) [file pone.0262471.s004.zip › S1-File-ORO/ORO/Fig3/HFD+E/4.tif]

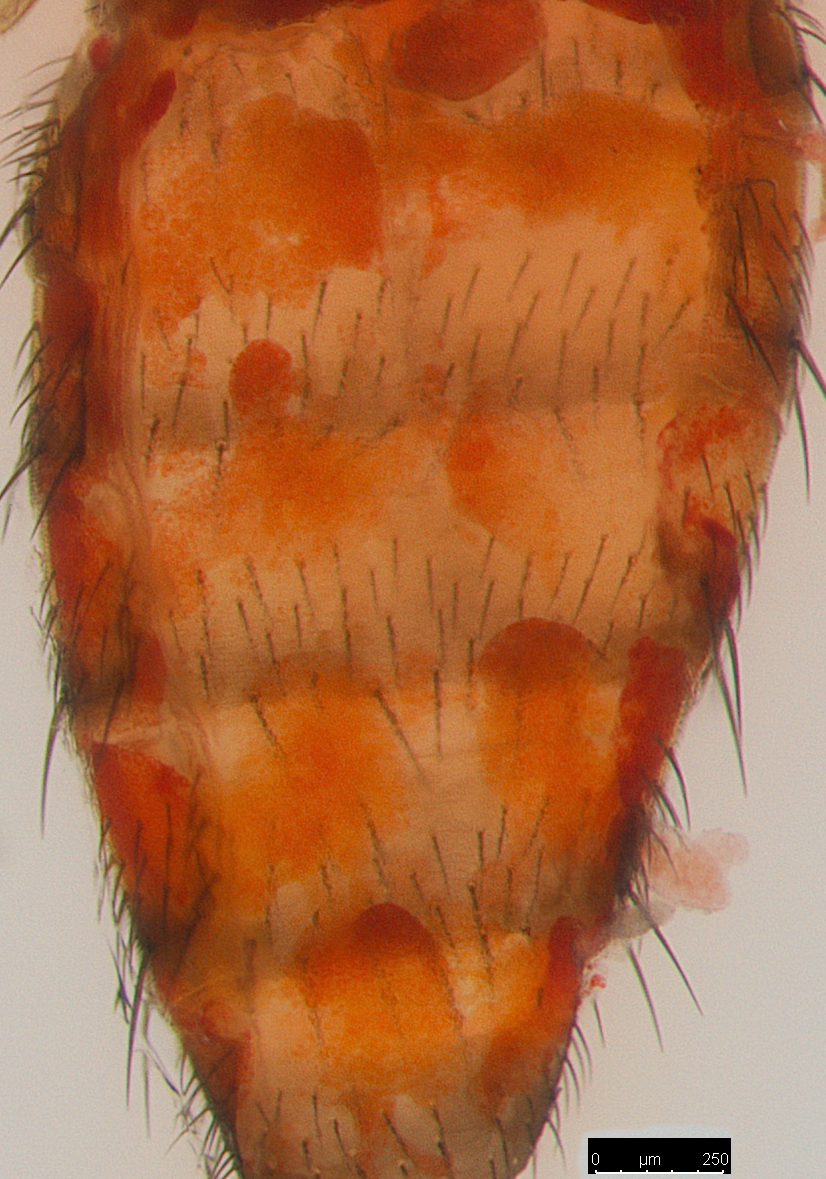

Supplement: S1 File — (ZIP) [file pone.0262471.s004.zip › S1-File-ORO/ORO/Fig3/HFD+E/HFD+E.tif]

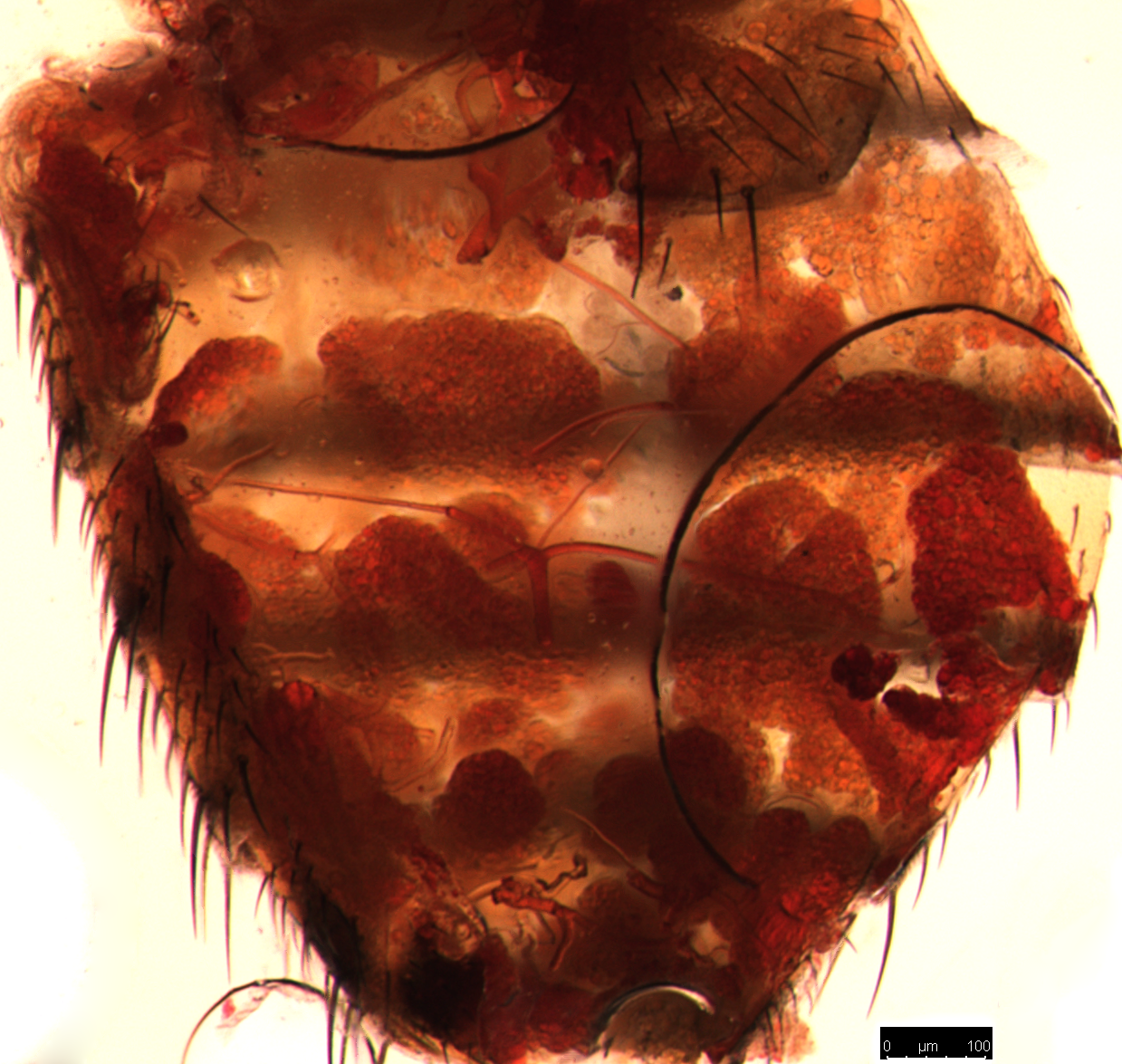

Supplement: S1 File — (ZIP) [file pone.0262471.s004.zip › S1-File-ORO/ORO/Fig3/HFD/1.tif]

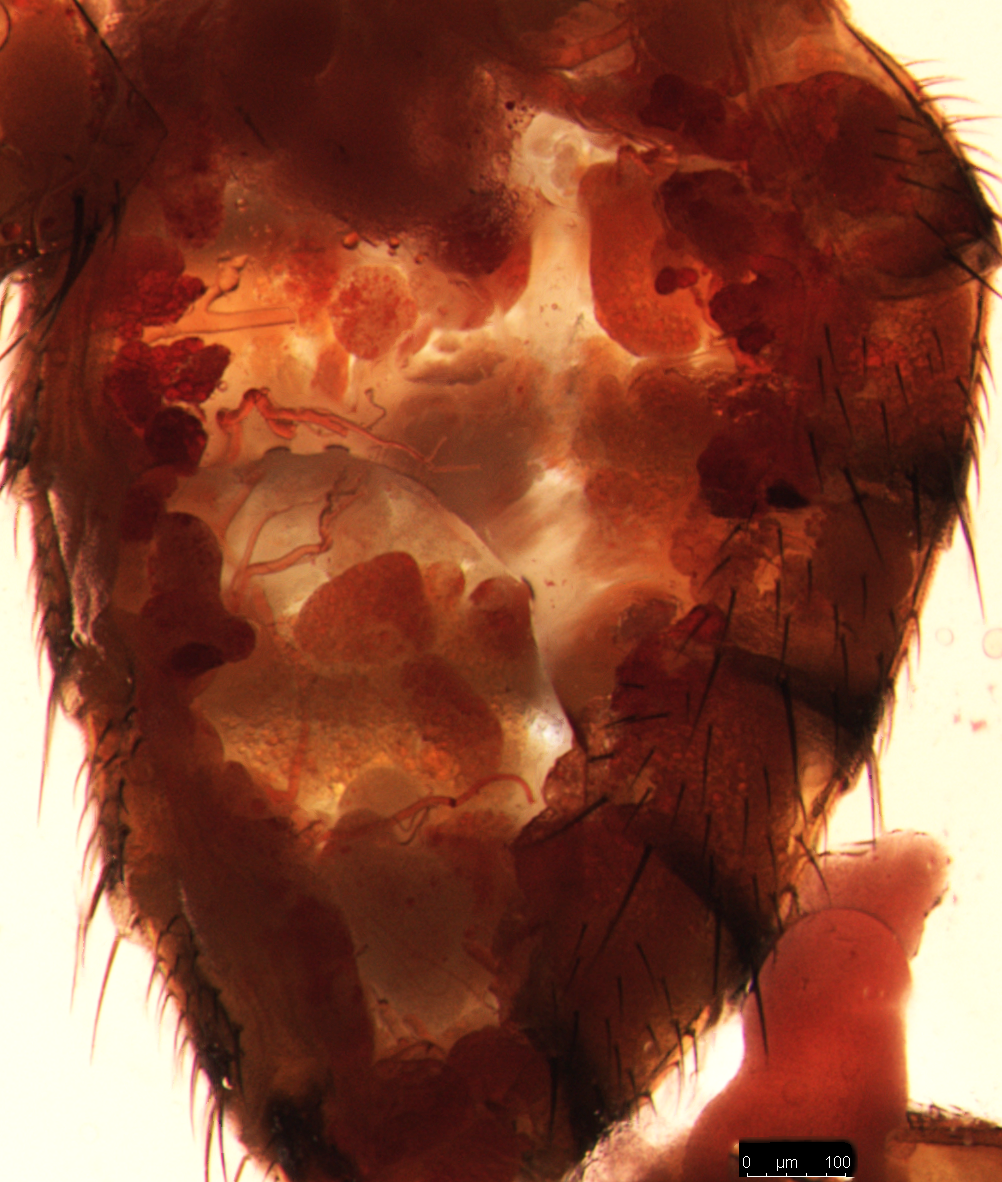

Supplement: S1 File — (ZIP) [file pone.0262471.s004.zip › S1-File-ORO/ORO/Fig3/HFD/2.tif]

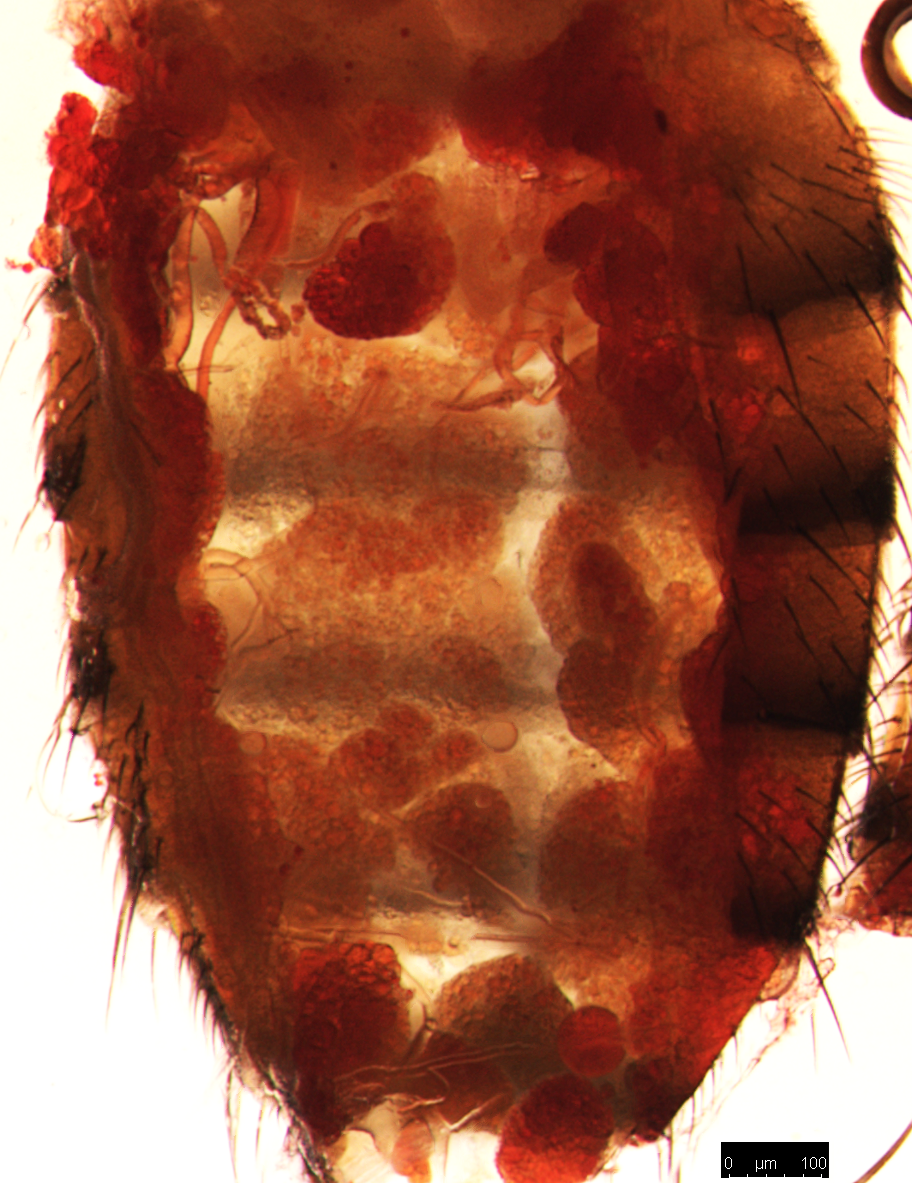

Supplement: S1 File — (ZIP) [file pone.0262471.s004.zip › S1-File-ORO/ORO/Fig3/HFD/3.tif]

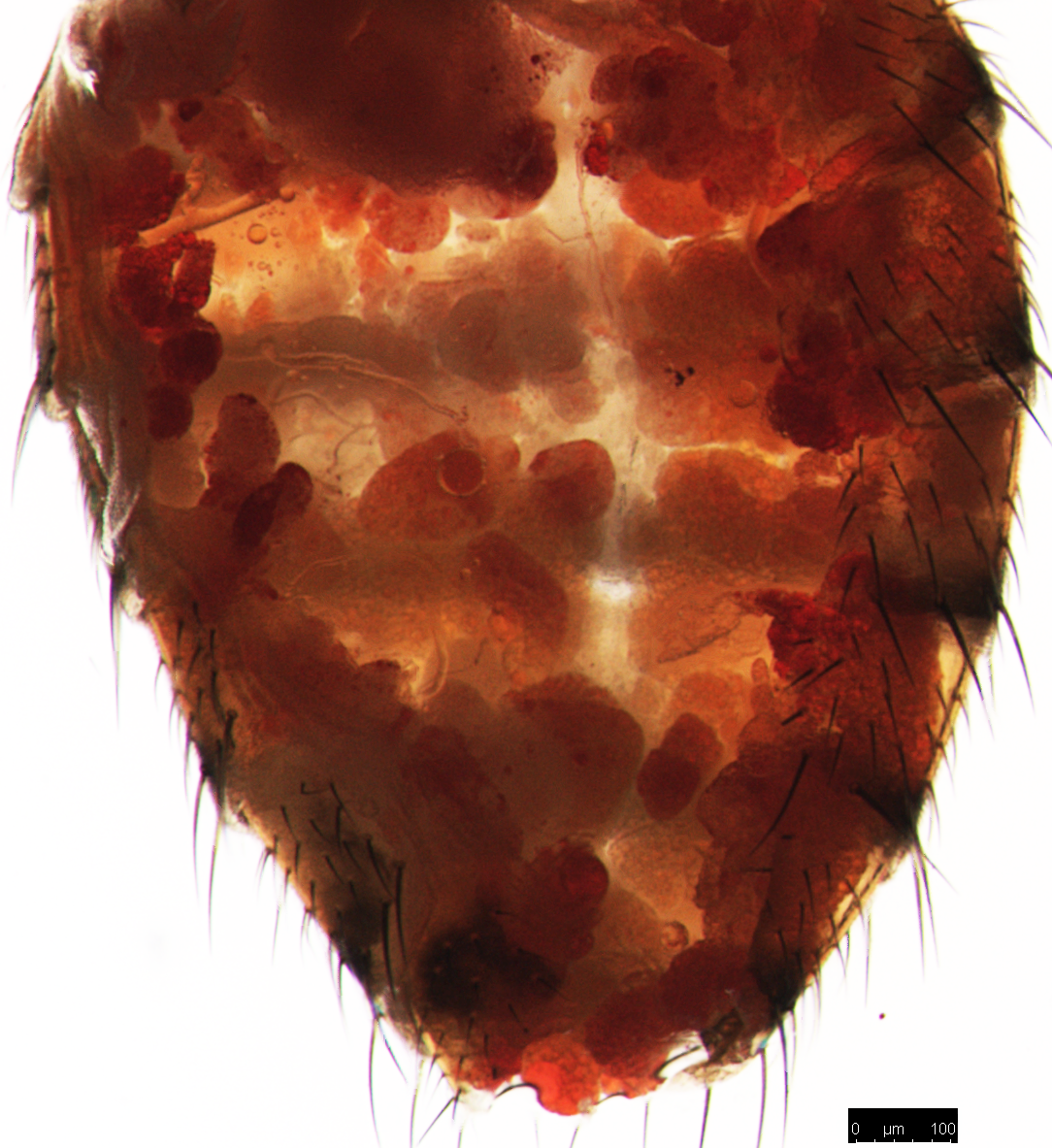

Supplement: S1 File — (ZIP) [file pone.0262471.s004.zip › S1-File-ORO/ORO/Fig3/HFD/4.tif]

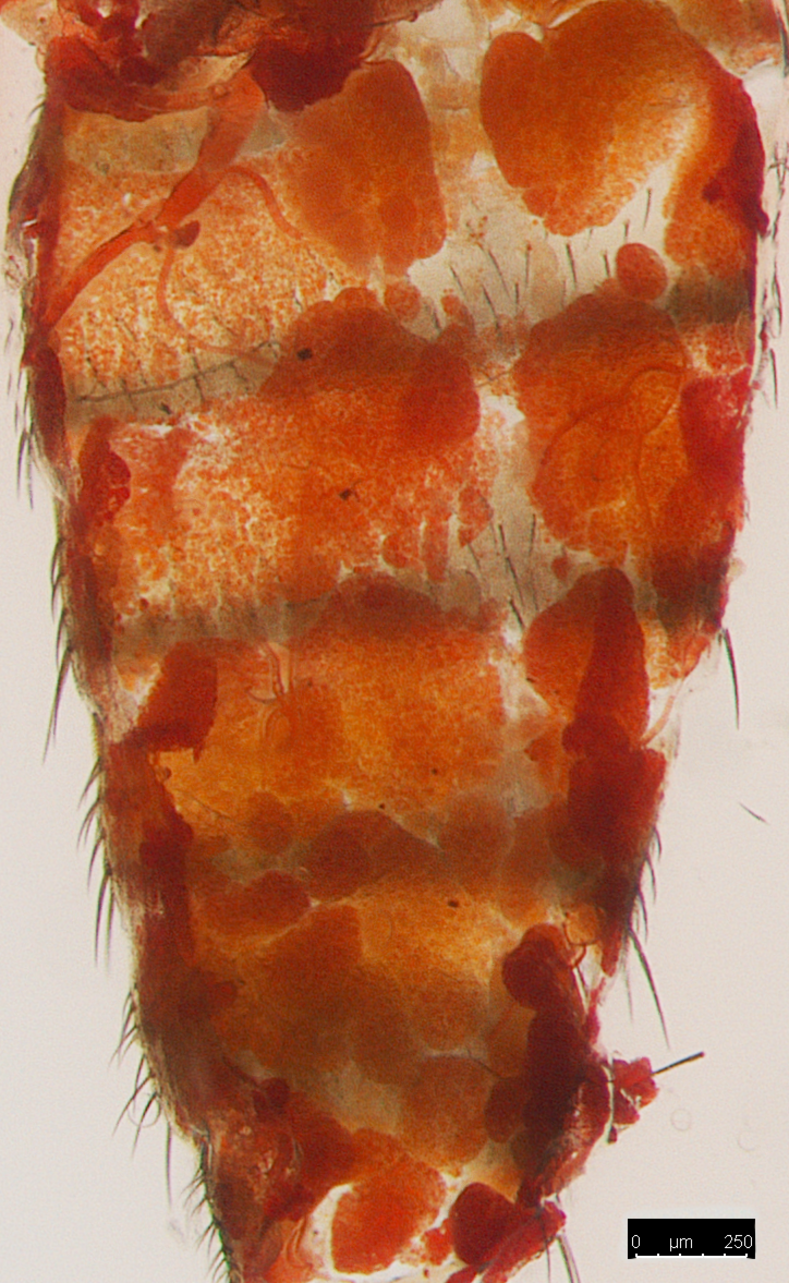

Supplement: S1 File — (ZIP) [file pone.0262471.s004.zip › S1-File-ORO/ORO/Fig3/HFD/HFD.tif]

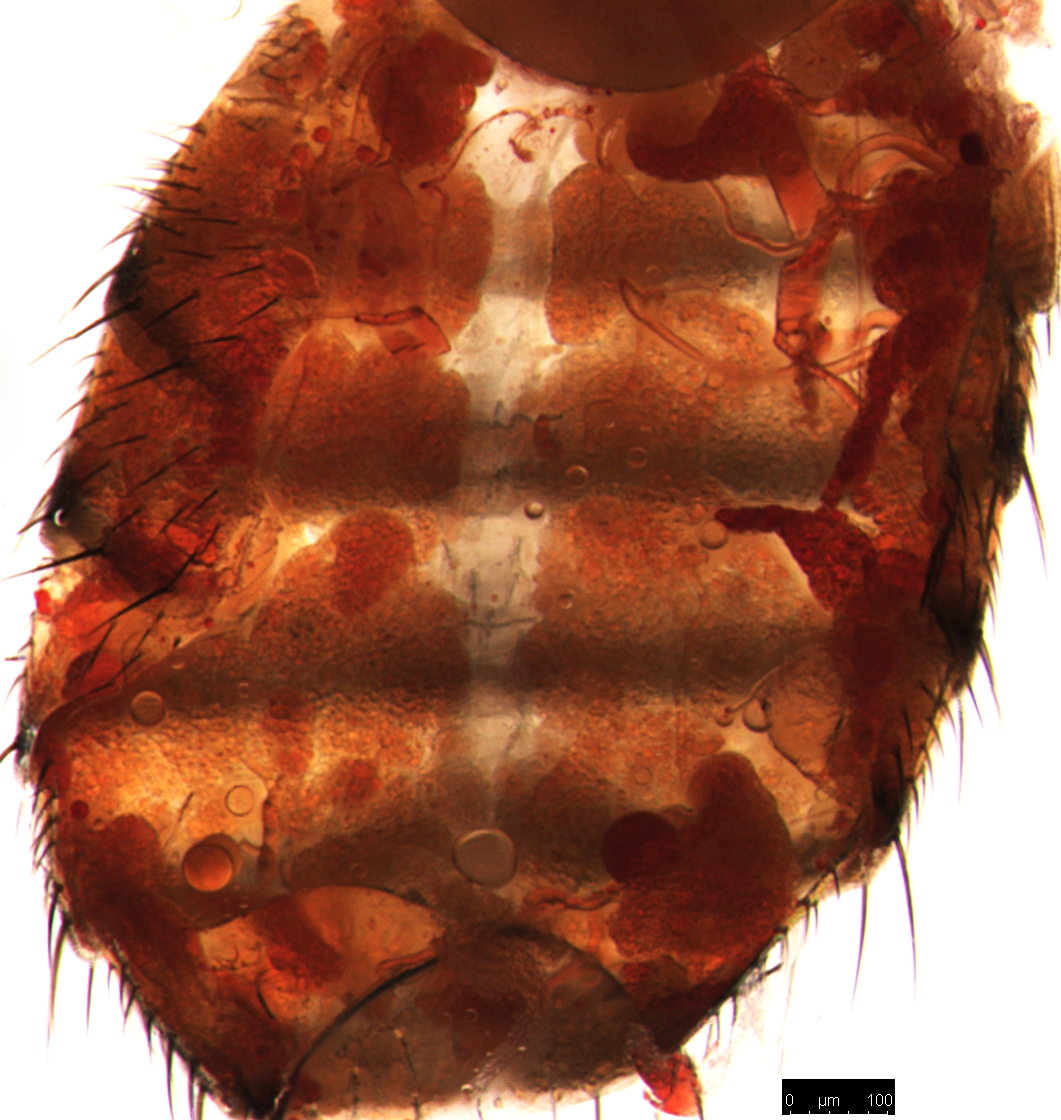

Supplement: S1 File — (ZIP) [file pone.0262471.s004.zip › S1-File-ORO/ORO/Fig4/HFD+E+KD/1.tif]

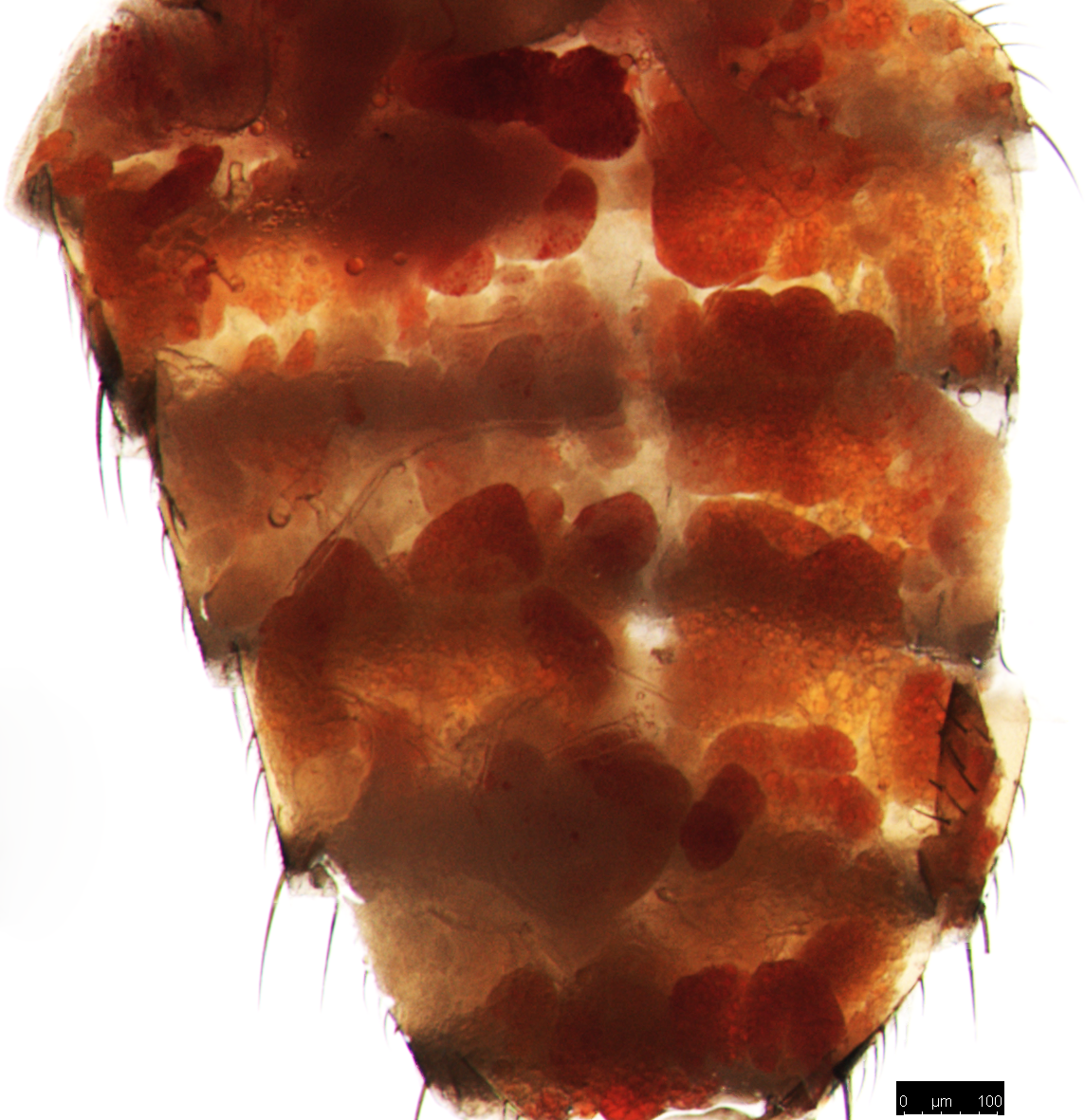

Supplement: S1 File — (ZIP) [file pone.0262471.s004.zip › S1-File-ORO/ORO/Fig4/HFD+E+KD/2.tif]

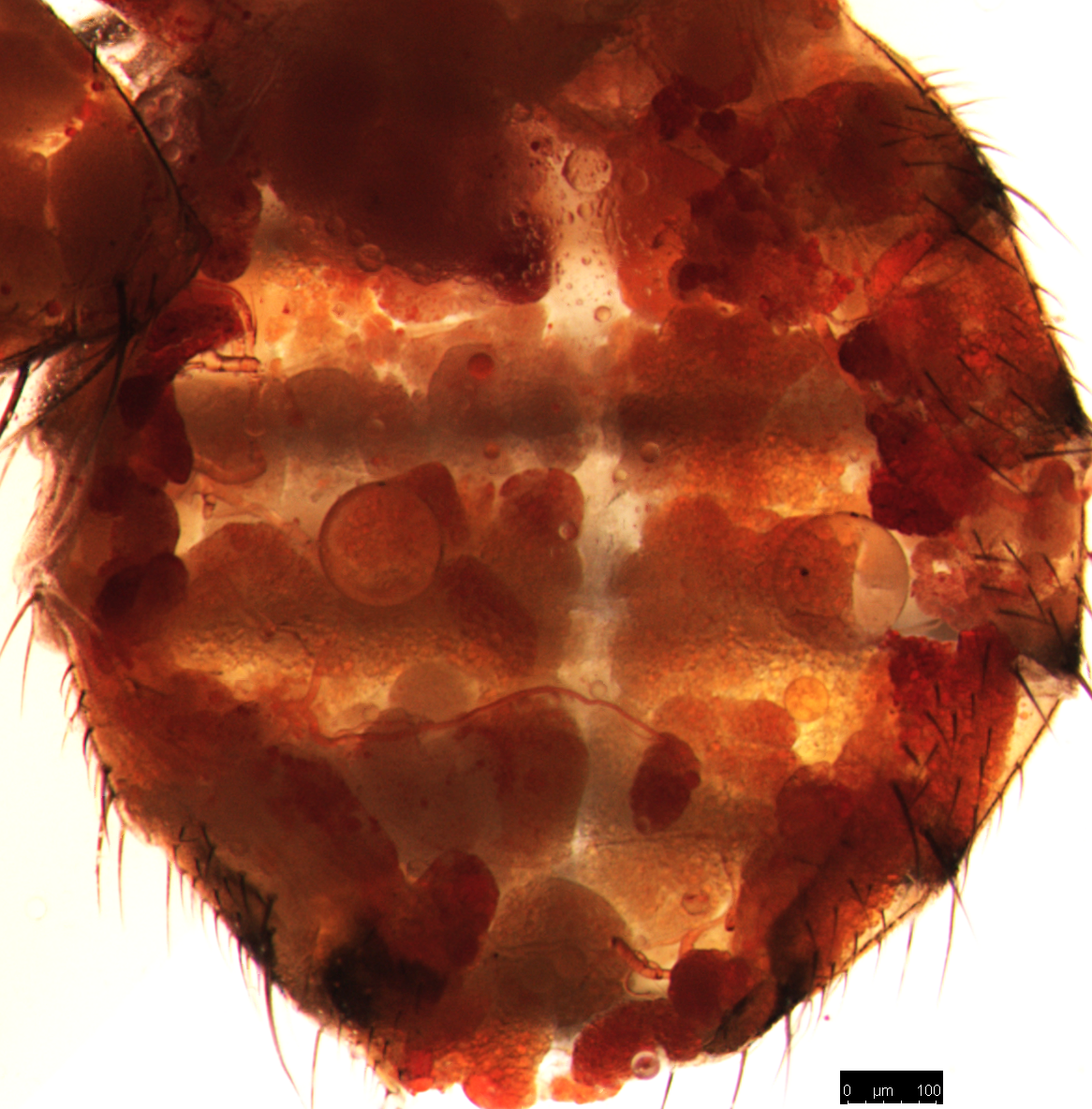

Supplement: S1 File — (ZIP) [file pone.0262471.s004.zip › S1-File-ORO/ORO/Fig4/HFD+E+KD/3.tif]

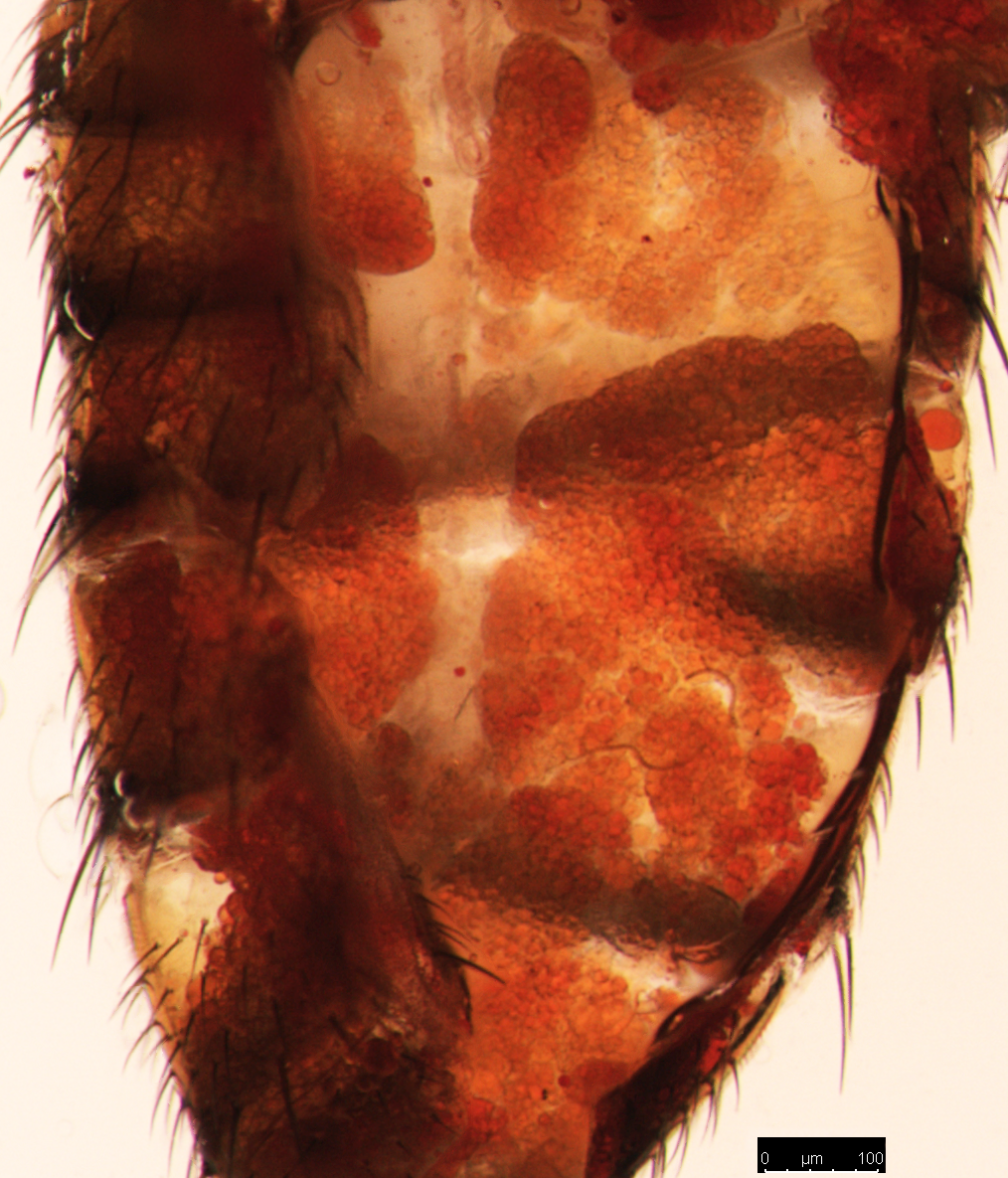

Supplement: S1 File — (ZIP) [file pone.0262471.s004.zip › S1-File-ORO/ORO/Fig4/HFD+E+KD/4.tif]

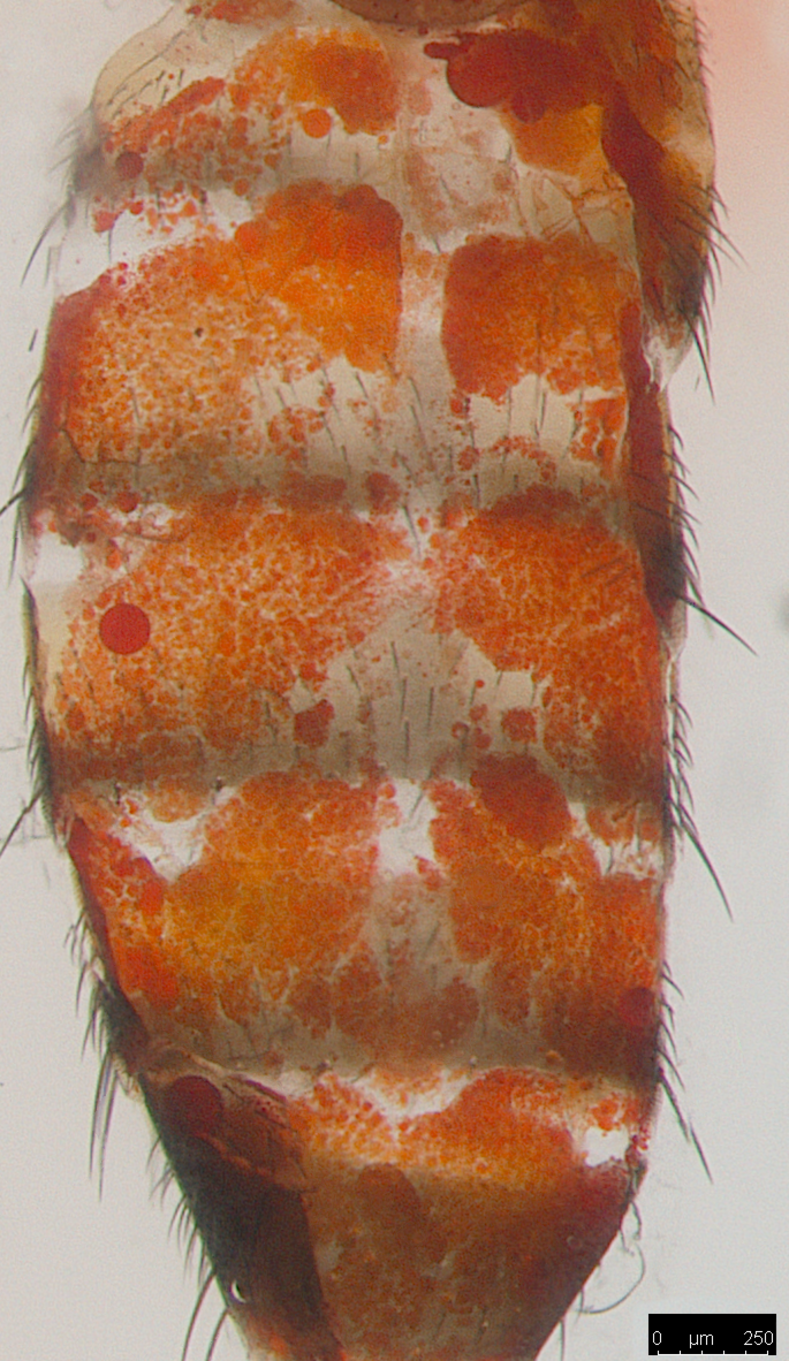

Supplement: S1 File — (ZIP) [file pone.0262471.s004.zip › S1-File-ORO/ORO/Fig4/HFD+E+KD/HE+KD.tif]

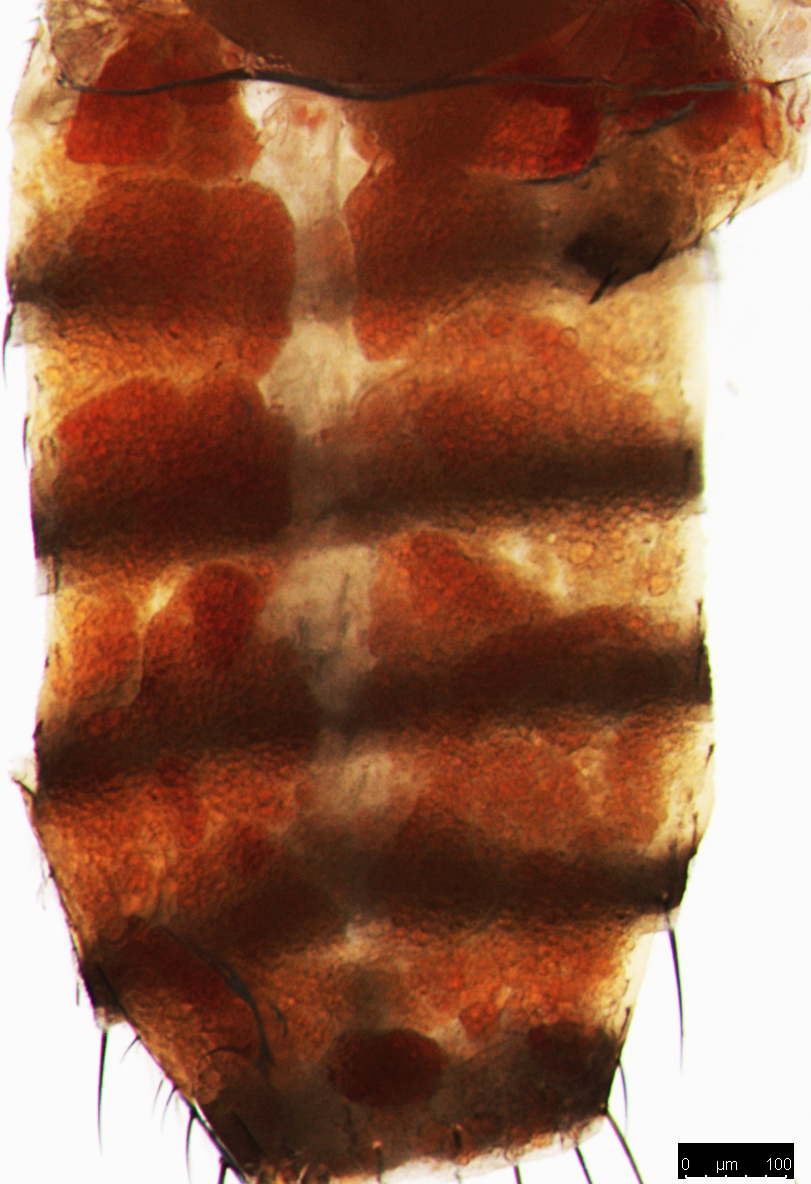

Supplement: S1 File — (ZIP) [file pone.0262471.s004.zip › S1-File-ORO/ORO/Fig4/HFD+KD/1.tif]

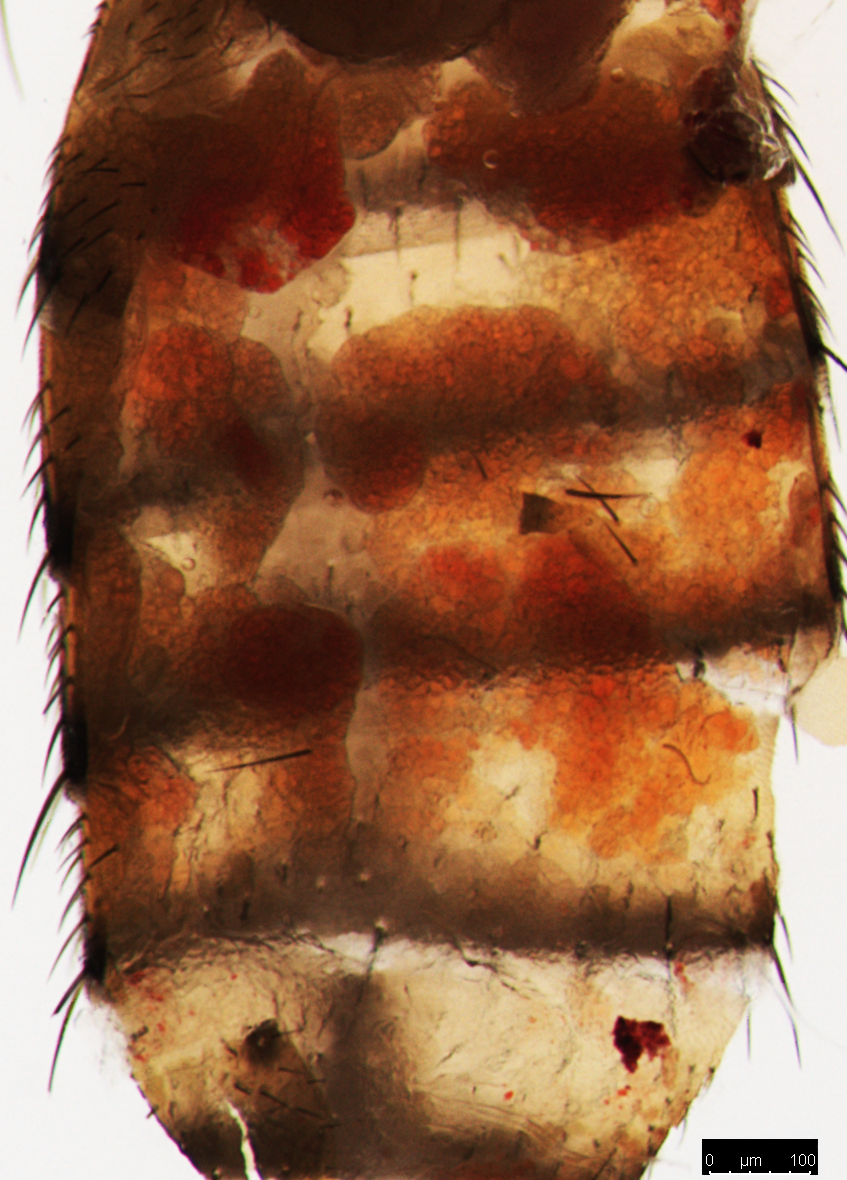

Supplement: S1 File — (ZIP) [file pone.0262471.s004.zip › S1-File-ORO/ORO/Fig4/HFD+KD/2.tif]

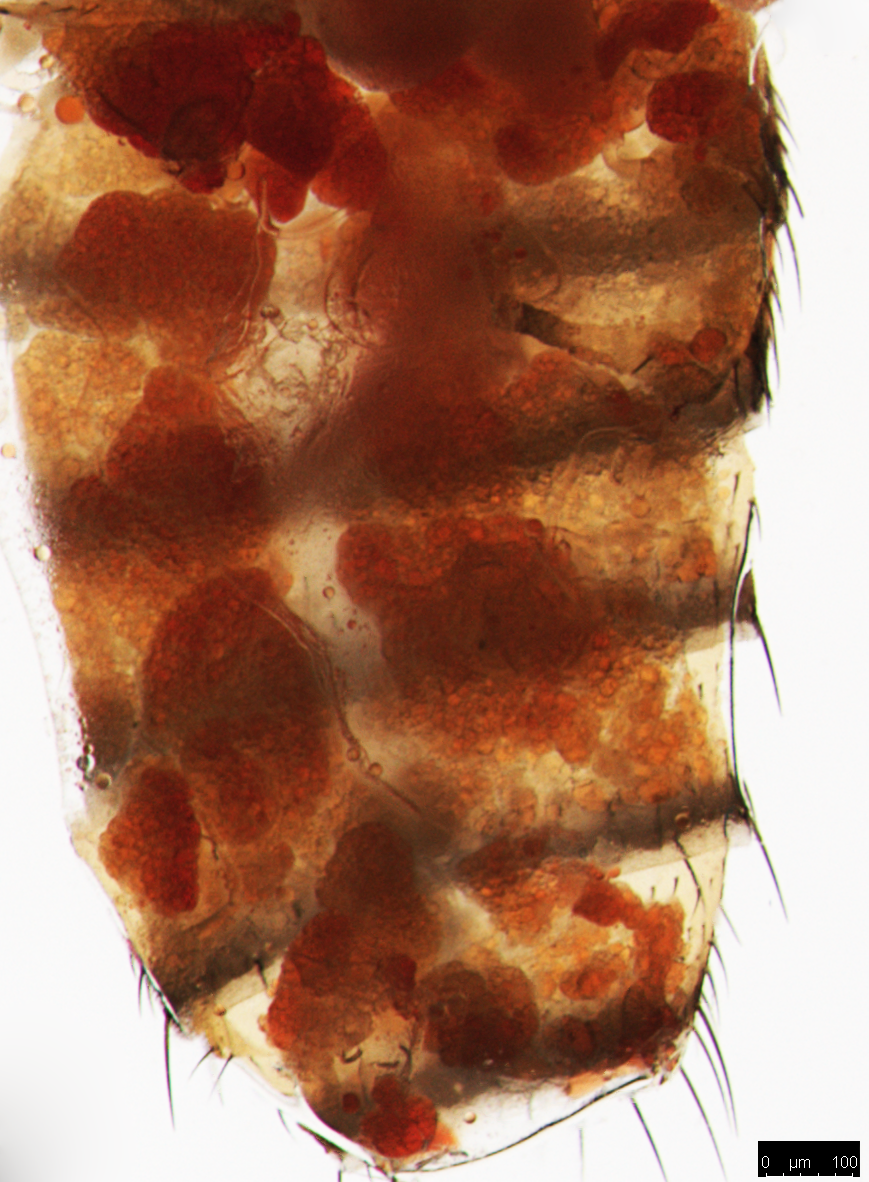

Supplement: S1 File — (ZIP) [file pone.0262471.s004.zip › S1-File-ORO/ORO/Fig4/HFD+KD/3.tif]

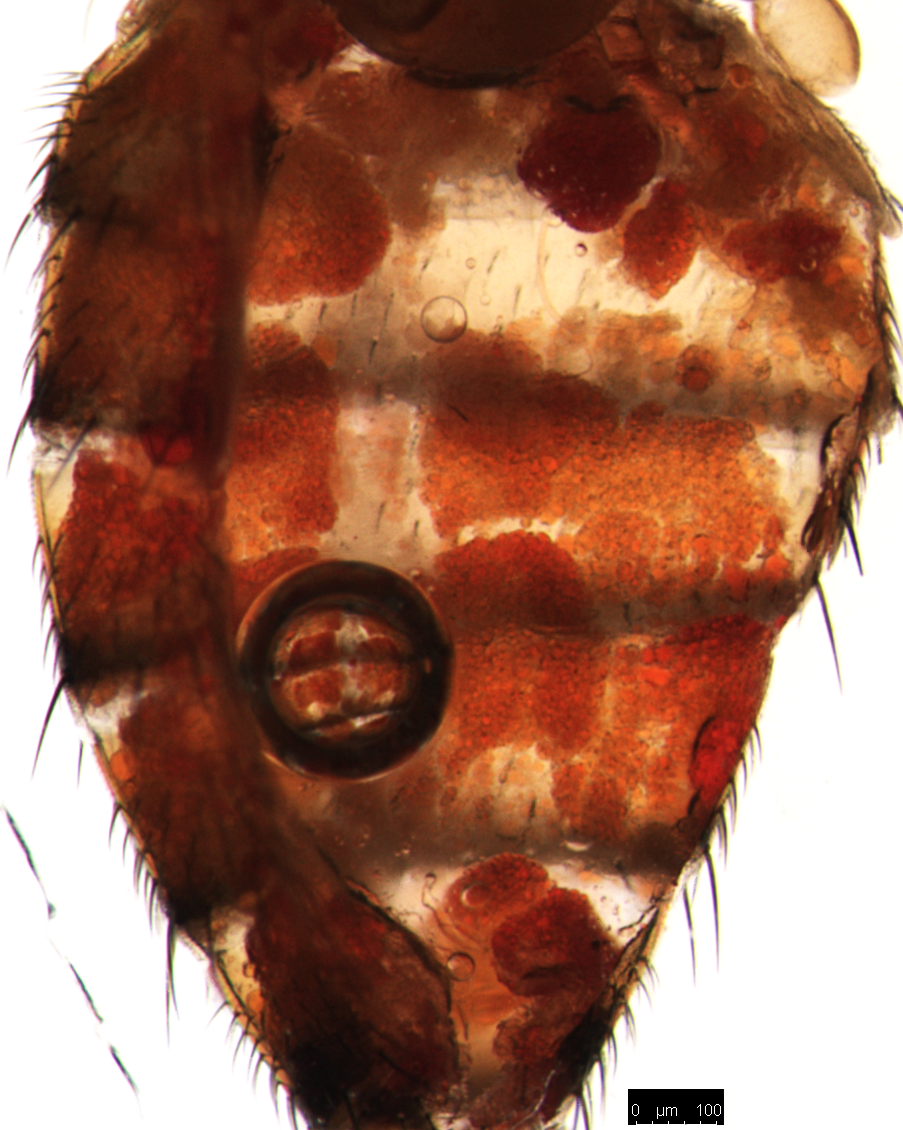

Supplement: S1 File — (ZIP) [file pone.0262471.s004.zip › S1-File-ORO/ORO/Fig4/HFD+KD/4.tif]

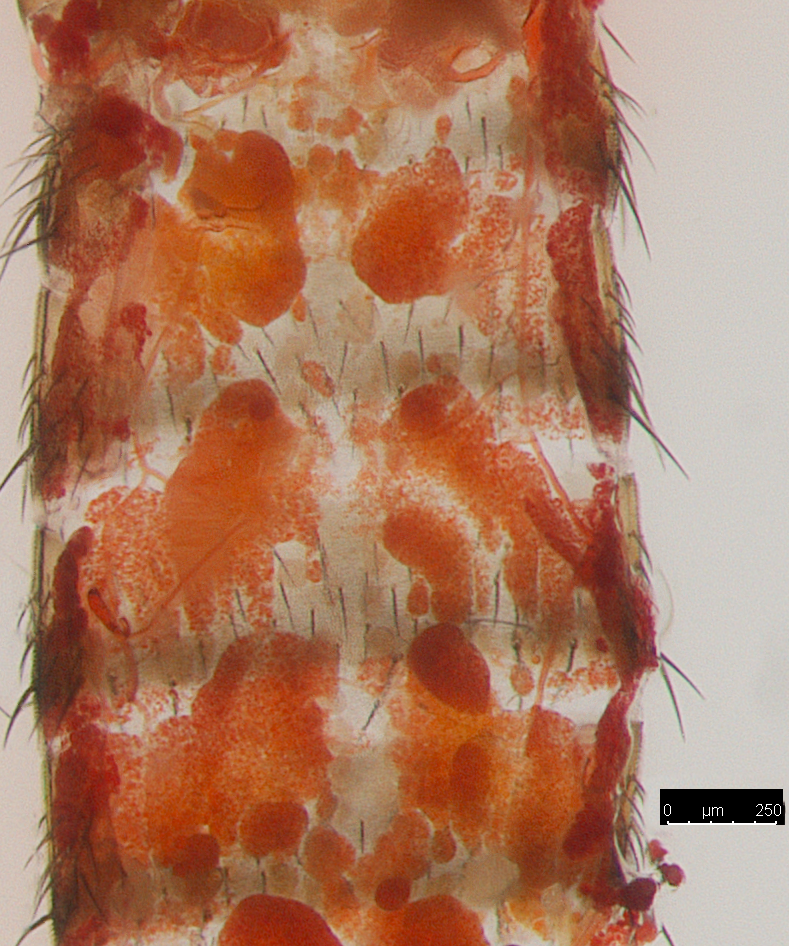

Supplement: S1 File — (ZIP) [file pone.0262471.s004.zip › S1-File-ORO/ORO/Fig4/HFD+KD/HFD+KD.tif]

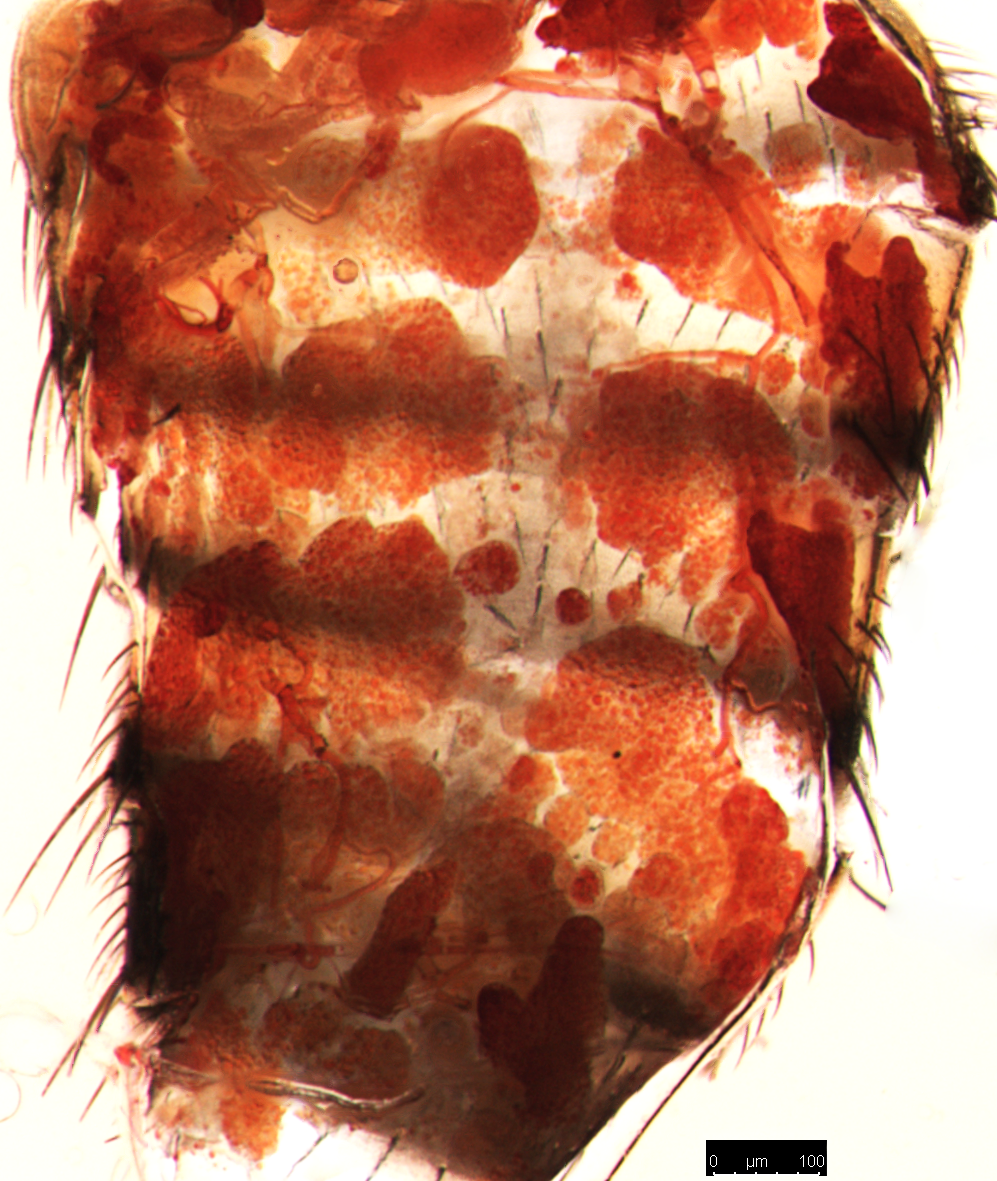

Supplement: S1 File — (ZIP) [file pone.0262471.s004.zip › S1-File-ORO/ORO/Fig4/NF/1.tif]

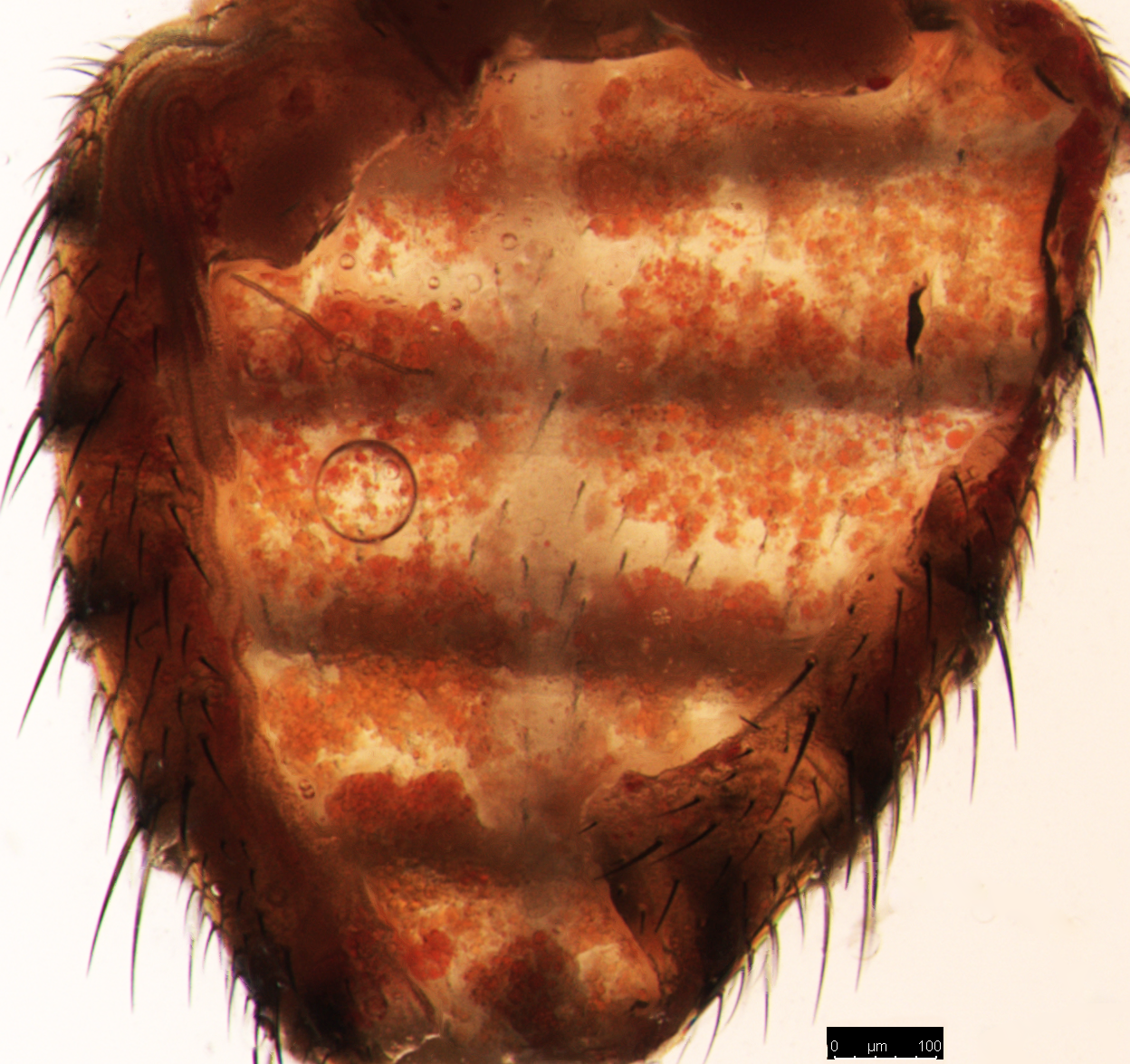

Supplement: S1 File — (ZIP) [file pone.0262471.s004.zip › S1-File-ORO/ORO/Fig4/NF/2.tif]

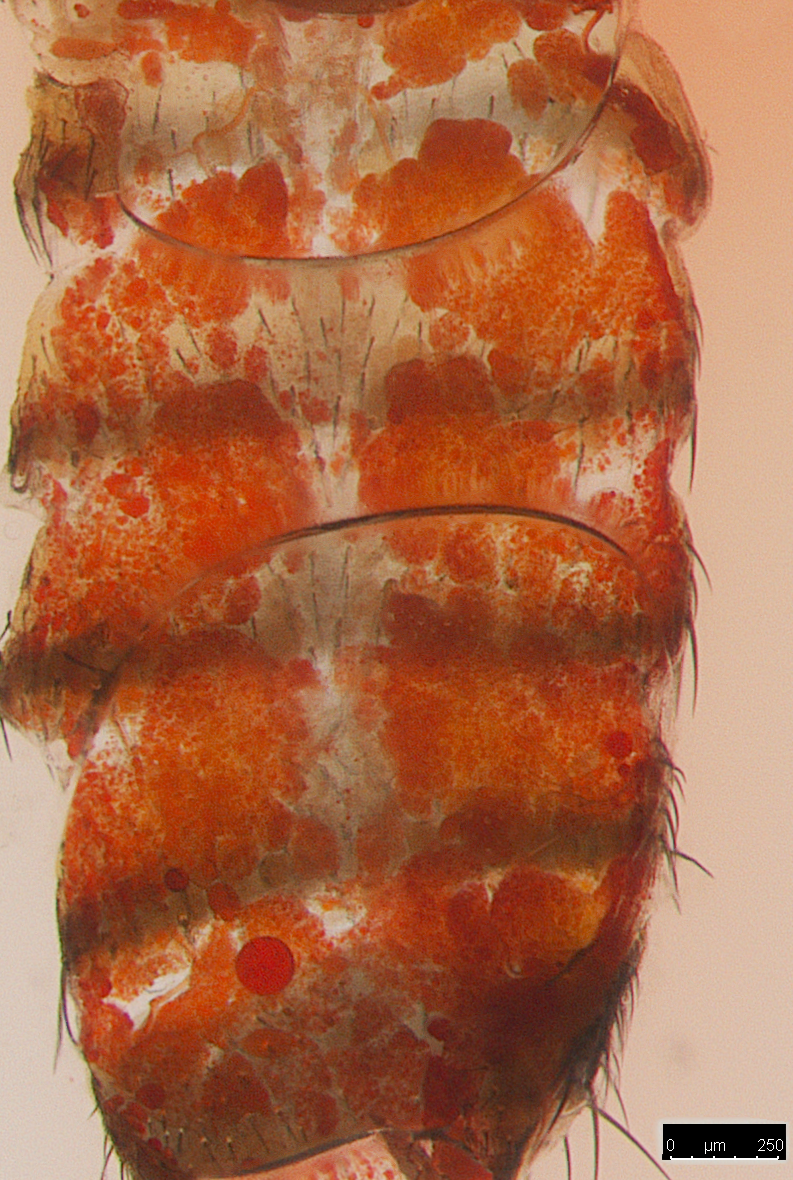

Supplement: S1 File — (ZIP) [file pone.0262471.s004.zip › S1-File-ORO/ORO/Fig4/NF/3.tif]

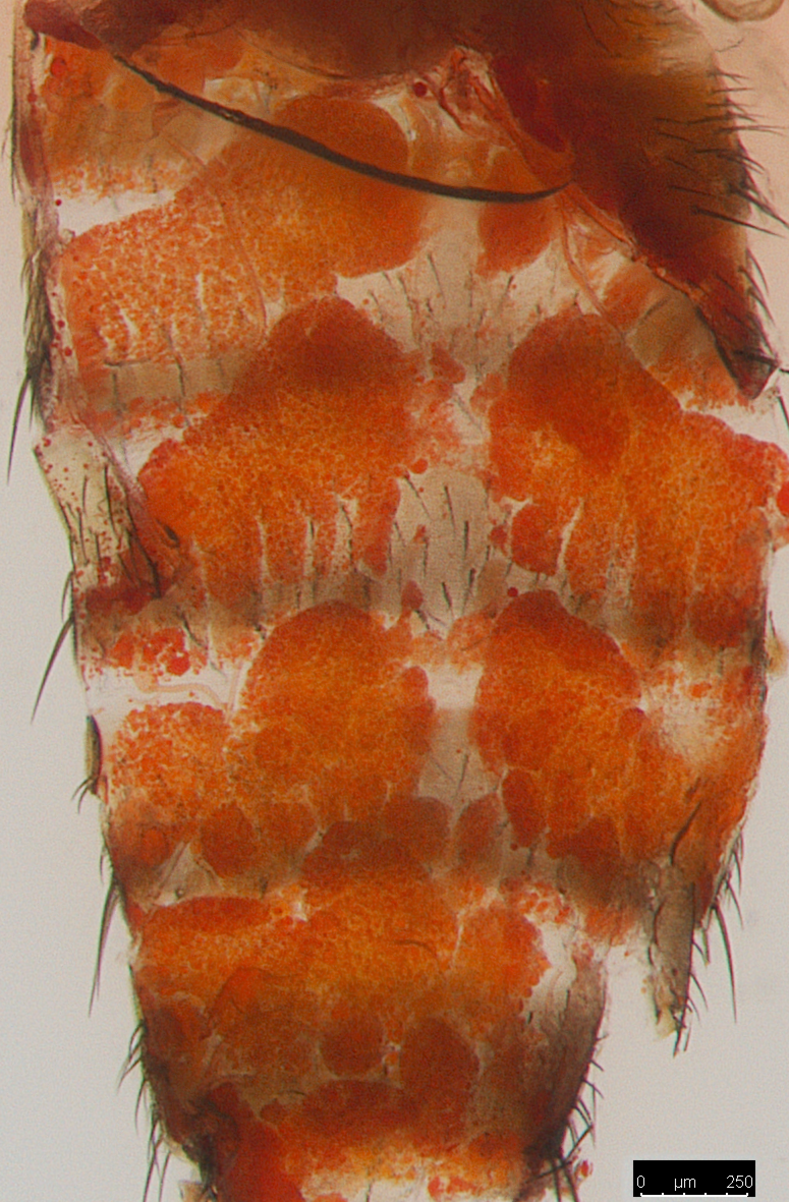

Supplement: S1 File — (ZIP) [file pone.0262471.s004.zip › S1-File-ORO/ORO/Fig4/NF/4.tif]

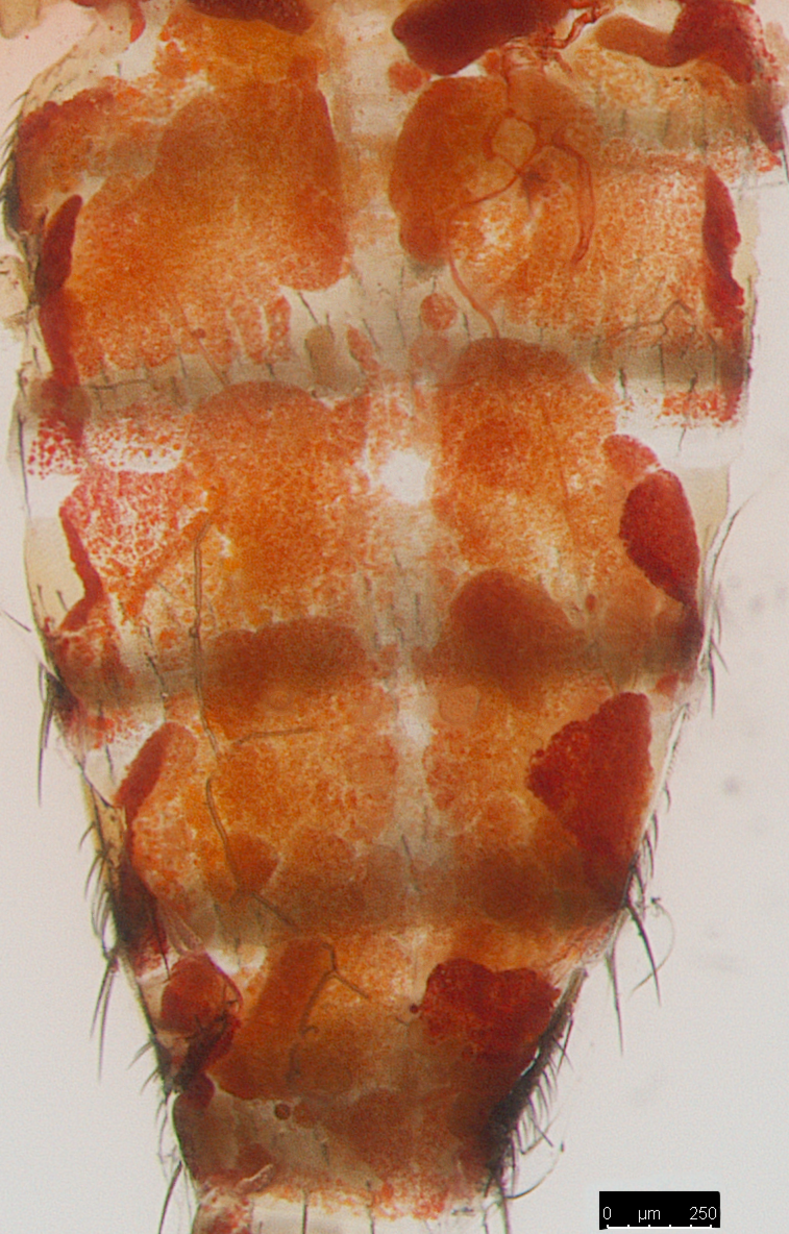

Supplement: S1 File — (ZIP) [file pone.0262471.s004.zip › S1-File-ORO/ORO/Fig4/NF/NF1.tif]
