# Supplementary material for: Effects of Drosophila melanogaster regular exercise and apolipoprotein B knockdown on abnormal heart rhythm induced by a high-fat diet
Source: PLoS One. 2022 Jun 3;17(6):e0262471. doi: 10.1371/journal.pone.0262471 (PMC9165823; doi:10.1371/journal.pone.0262471)
Supplement: Attachment — Submitted filename: Response to Reviewers.docx [file pone.0262471.s005.docx]

Dear Dr. Girish C. Melkani,

Thank you for your letter and for the reviewers' comments concerning our manuscript entitled "Effects of Drosophila melanogaster regular exercise and apolipoprotein B knockdown on abnormal heart rhythm induced by a high-fat diet". Your comments and those of the reviewers were highly insightful and enabled us to greatly improve the quality of our manuscript. In the following pages are our point-by-point responses to each of the comments of the reviewers as well as your own comments.

Thank you and best regards.

Yours sincerely,

First author: Meng Ding

Corresponding author: Lan Zheng

E-mail: [lanzheng@hunnu.edu.cn](mailto:lanzheng@hunnu.edu.cn)

**List of Responses**

**Responds to the editor's comments:**

1. We have re-checked the references in accordance with the requirements of the journal. We deleted a retracted paper and found a replacement.

**Responds to the reviewer’s comments:**

**Reviewer #1:**

Q1. My concern about feeding rate was not a concern about how the different groups were housed and fed, it was a concern about whether HFD or exercise themselves would alter feeding rate, which was not tested here, and would be a good control to add in future. Authors could at least reference other studies that have shown those controls, which may be good enough here.

A1. We have indeed neglected to control the feeding rate. At first, the establishment of the high-fat model was based on the research of Bird RT, 2010. In more than ten years of progress, the control of the feeding behavior of flies has become stricter, because the intake of the right type and amount of food has a major impact on the quality of life. In the future, we will focus on controlling the feeding rate of flies in order to obtain more convincing data. In addition, we explained this in the manuscript, and the specific content is highlighted in red.

Q2. The only change that I would insist on at this point is that the authors need to write their conclusions without stating that exercise acts through apoLpp to exert its effects on heart function and fat storage. All of these experiments are also consistent with these two things acting in parallel, and this is a key point. Just because exercise reduces apoLpp levels does not mean that this is the key mechanism of exercise effects. Authors should say something more like "apoLpp reduction may be involved in exercise-induced protection against HFD".

A2. The conclusion is indeed not concise and accurate enough. We very much agree with the reviewer's point of view and have made corresponding amendments in the conclusion.

**Reviewer #2:**

Q1. Line 68 “sufficiently reduced to resist HFD-induced cardiac function impairment” is confusing. I think instead of resist they might mean elicit or potentiate.

A1. I read the original text again and revised this sentence.

"Fibrillation of the Drosophila heart may result from lipotoxic damage related to the insulin-TOR signal, which is moderate reduction in insulin-TOR signaling prevents HFD-induced obesity and cardiac dysfunction"

Q2. Line 87: w1118 has a lower case w.

A2. We made a change to this and highlighted it in red in the text.

Q3. Line 146. This TG assay is still of concern and my previous concern was not addressed. TG ELISAs seem to measure thyroglobulin, not triglycerides. There are not enough details to ascertain what was done. I do not believe triglycerides can be measured by an ELISA and the authors have given me no information to believe otherwise. A Google search for the kit listed produces nothing. What was used as the standard? Figs 1H and 2H should have a unit (ug TG/mg fly or something like this) not OD. OD isn’t as informative.

A3. Thank you very much for your reminder. We realize that ELISA is not an effective way to quantify TG levels compared with the use of biochemical methods. We have added detailed experimental steps in Materials and Methods. In future research, we will circumvent this problem and choose more suitable experimental methods to quantify the data. In addition, we are willing to provide all the information on the kit for reviewers to view. The kit (Insect TG ELISA Kit) we use is produced by Shanghai Enzyme-linked Biotechnology, https://www.mlbio.cn/. We upload the kit instructions with the manuscript.

Q4. Line 164: How was Photoshop used to quantify the intensity of staining? Was a thresholding done to identify the red areas of the fat body cells? Were these dissociated from the cuticle before mounting and imaging? Some description would be helpful here.

A4. We added detailed methods in the manuscript. Invert the color image into a black/white image to identify the stained area, and use the average pixel density to quantify the staining intensity. In addition, before installation and imaging, the damage to adipose tissue should be reduced as much as possible to ensure the accuracy of the data. All changes are highlighted in red font in the materials and methods section.

Q5. Line 206: Figure 1: was this data generated in the control, transheterozygous genotype (UAS-Lpp RNAi x w1118 offspring)?

A5. Yes, we have rewritten the title of Figure 1 to make it more clear. “*UAS-apoLpp RNAi*>W^1118^ group was exposed to HFD for 5 days, resulting in obesity and abnormal heart rhythm”

Q6. Line 288: The figure title is not fixed as the authors say in the response. The old, bad title is retained in the manuscript. Please use the new, improved title that you wrote in the reviewer response.

A6. I personally express my apologies, this is a mistake. I have replaced the title of Figure 3 with the latest version.

Q7. Line 298-300: the G and H descriptions need to be switched to match the figure.

A7. The G and H in Figure 3 have been modified to their correct positions.

Q8. Line 331- Figure 4: it doesn’t make sense to me to exclusively compare the normal diet control genotype to the HFD knockdown genotype, although I appreciate the fact that they are so close to each other. It seems as though the HFD control genotype should be included so the reader can see what knockdown and exercise does to each genotype. I realize this may be duplicative of the data shown in other panels, but if possible, it’s easiest to see differences when data are compared side-by-side.

A8. In order to solve this problem, we reformatted Figure 4 and added the HFD control group on the original basis. As the reviewer said, this combination makes it easier to see the difference.

Q9. Line 333: superimposed effects could read “combined effect”

A9. I agree with your point of view and made corresponding changes in the manuscript.

Q10. Line 393: it reads “Knockdown of apoLpp in cardiomyocytes… does not directly affect the heart” I do not think this conclusion can be made given the data shown.

A10. This is a wrong conclusion, and we have deleted it from the manuscript. It should have been deleted in the first revision, but it was accidentally retained due to our negligence.

Q11. Line 400: reads “Flies allowed to exercise regularly show similar results to mammals with regard to high-fat- or high-sugar-induced heart damage.” The references provided do not seem to show these results.

A11. We re-read the cited article and found that this sentence is indeed an extension. We rewritten the sentences and added new quotes.

Q12. The tracked changes would be easier for me to read without the old text in place; it’s easiest if the replacement text is highlighted.

A12. We upload the manuscript, the manuscript with tracking changes, and the instructions for the ELISA kit to PLOS ONE.
